# Human-induced pressures and environmental factors drive the distribution of neglected tropical disease Buruli ulcer

**DOI:** 10.1038/s43856-026-01692-6

**Published:** 2026-06-04

**Authors:** Alisa Aliaga-Samanez, Marina Cobos-Mayo, Christine Chevillon, Magdalene Dogbe, Kayla M. Fast, Matthew E. Scott, Kelly Waters, Joseph P. Receveur, Jennifer L. Pechal, Michael W. Sandel, Heather R. Jordan, M. Eric Benbow, Jean-François Guégan

**Affiliations:** 1 MIVEGEC (UMR Université de Montpellier, CNRS, IRD), Montpellier, France; 2 Instituto de Investigación en Recursos Cinegéticos (IREC) – CSIC, UCLM, JCCM, Ciudad Real, Spain; 3 Grupo de Biogeografía, Diversidad y Conservación, Departamento de Biología Animal, Facultad de Ciencias, Universidad de Málaga, Málaga, España; 4 Department of Biological Sciences, Mississippi State University, Mississippi State, MS USA; 5 Department of Wildlife, Fisheries and Aquaculture, Mississippi State University, Mississippi State, MS USA; 6 Department of Entomology, Michigan State University, East Lansing, MI USA; 7 Institute for Genome Sciences, University of Maryland Baltimore, Baltimore, MD USA; 8 Forest and Wildlife Research Center, Mississippi State University, Mississippi State, MS USA; 9 Department of Osteopathic Medical Specialties, Michigan State University, East Lansing, MI USA; 10 Ecology, Evolution and Behavior Program, Michigan State University, East Lansing, MI USA; 11 AgBioResearch, Michigan State University, East Lansing, MI USA

**Keywords:** Infectious diseases, Microbiology

## Abstract

**Background:**

Understanding the spatial distribution of skin neglected tropical diseases (NTDs) is paramount for surveillance and control strategies, although little is known regarding their natural origin, ecology, and distribution. This study aims to determine how natural and anthropogenic factors affect local to global spatial distribution patterns of a major skin NTD, Buruli ulcer (BU), and its pathogen, *Mycobacterium ulcerans* (MU).

**Methods:**

Generalized linear modelling of 110 variables grouped into five categories (i.e., physical, edaphic/climatic, animal diversity, plant diversity, and socio-demographic/geographic) were used to estimate continental-scale patterns of both MU and BU cases in Africa. Spatial models allowed the production of BU and MU maps for tropical and subtropical zones worldwide.

**Results:**

Natural factors and their interactions consistently influence MU and BU distributions in Africa, with these distributions also responding to anthropogenic factors such as invasive plant species, agriculture and other activities. BU case distribution was driven by sunlight radiation, especially UV-B seasonality. Our extrapolation maps provide a quantitative understanding of the potential distribution of this pathogen and associated BU disease burden.

**Discussion:**

Our findings highlight the utility of a disease ecology perspective using large-scale spatial data for understanding drivers of skin NTDs. Complex interactions between natural and anthropogenic factors indicate that developing preventive and control measures is challenging. Particular attention is needed on the role of invasive plant species in the spread of these pathogens, and the complex influence of sun exposure on skin NTDs.

## Plain language summary

The ecological forces shaping neglected tropical diseases (NTDs) remain largely unknown. Buruli ulcer, a debilitating skin NTD, offers a striking case to explore how climate, biodiversity, and human activities interact together to drive NTD emergence and spread. Why does its spatial distribution align with some tropical regions but not others? What factors explain its geographical distribution across the tropics? Our analyses reveal that disease patterns reflect unexpected drivers, from presence of invasive plant species to seasonal sun exposure, alongside agriculture development and other human pressures. Extrapolated globally, these patterns highlight potential disease hotspots across the intertropical zones, with potential extension outside the tropics. By exposing how natural and human-derived factors converge to shape disease distribution, our work underscores the need for disease ecological perspectives in public health and offers a framework to anticipate emerging NTD risks.

## Abstract (web summary)

Aliaga-Samanez et al. examine the spatial distribution of Buruli ulcer and its Mycobacterium ulcerans pathogen using physical, edaphic, climatic, animal and plant diversity, socio-demographic, and geographic factors. Their findings show that interacting natural and anthropogenic drivers shape disease patterns and complicate prevention and control strategies.

## Introduction

Skin-related neglected tropical diseases (skin NTDs), including leprosy, cutaneous leishmaniasis, yaws (or pian, pinta, bejel), and Buruli ulcer, impact humans through stigma and social exclusion^1^. Globally, skin NTDs are the fourth leading cause of human disease and 18th for disability-adjusted life years (DALY)^2^. Common features among multiple skin NTDs highlight the utility of shared care strategies^3,4^. Such an approach is relatively new, and draws on older paradigms, which is different from current disease-specific approaches, of pathocœnosis (stipulating that diseases do not occur in isolation but through dynamic interactions with environmental, social, and cultural factors) and can benefit from an ecosystem-based framework^5^. The 78th World Health Assembly recently adopted the resolution “Skin Diseases as a Global Public Health Priority”^6^, highlighting the growing recognition of the importance of integrating skin NTDs into global health agendas.

Considerable research has addressed drivers responsible for emerging infectious diseases (EIDs) through the adoption of a disease ecology and pathogeography approach^7^. Environmental and socio-economic conditions (e.g., climate, land-use change, human-wildlife interactions, human demographics and behaviours, poverty and inequality), and their interactions, are inherently complex and intricate^8^. Species distribution and ecological niche models are computational tools used to characterize geographical distribution and spatial relationships of natural environmental conditions with a given animal or plant species occurrence^9^. They enable the construction of pathogen and disease geographical distribution maps based on probability values. For instance, Geng and colleagues^10^, used such models to determine that both climate and anthropogenic factors were major drivers of the global distribution of human bacterial infections.

However, there is little research using spatial modelling to understand sapronotic pathogens - those of environmental origin - that can notably cause skin NTDs. These pathogens, which persist in aquatic or terrestrial environments, remain poorly understood in terms of their ecological niches and transmission pathways^11^. Skin NTDs of sapronotic origin are often omitted from spatial analyses, limiting their ecological understanding and hampering control strategies^12^. Bridging this gap requires an integrative approach that combines ecological niche modelling with disease-specific insights.

This study provides a comprehensive understanding of patterns and processes involved in a naturally acquired skin NTD called Buruli ulcer (BU) disease, an infection due to a non-tuberculous bacillus, *Mycobacterium ulcerans* (MU). We used a comprehensive set of variables grouped into five categories (physical, edaphic/climatic, plant diversity, animal diversity, socio-demographic/geographic) to model the occurrence of both MU and BU across the African continent. This study is novel compared to previous work, particularly that of Simpson and colleagues^13^, in that it uses a much larger number of potentially explanatory factors and their interactions, based on a priori transmission hypotheses and which was not explicitly the case in previous studies. In addition, very limited work has been done on the regional and continental distribution of MU and BU^13^, most of investigations concentrating on local to supra-local scales^14,15^, thus avoiding potential influence exerted by medium- to large scale parameters. From a more general point of view, when such studies do exist, they often estimate MU and/or BU environmental suitability without identifying its main local to more global drivers.

## Methods

### Data source

Data on MU presence (in water and soil) and human BU case occurrence in Africa were obtained from publicly available sources, mainly from Simpson et al. (2019)^16^, and were complemented with additional published scientific articles (For more details on the data sources, see Supplementary Information Table S3). Among the data collected in Simpson et al.’ work^16^, we only considered MU as present when a sample was PCR positive for KR (ketoreductase enzyme activity) and/or ER (enoyl reductase activity). Likewise, we included only BU human cases when confirmed by positive PCR.

We used a total of 110 explanatory variables nested within the following categories: physical, edaphic and climatic, plant diversity, animal diversity, and geographic and socio-demographic. For details about the source and description of the different variables, see Supplementary information Table S4. We latticed the data using a grid in Africa consisting of 4061 hexagonal units of 88×88 km^2^, constructed using Discrete Global for *R* software (*R* Core team 2021) (https://github.com/r-barnes/dggridR). We only represented on the hexagonal lattice MU presence and BU cases that had a precise location or referred to an administrative unit smaller or similar in size to the hexagons. This dataset was transformed into binary variables; hereafter representing the distribution of MU and BU cases.

### Pathogeographical approach for the African continent and training models

**Fig. 1:**
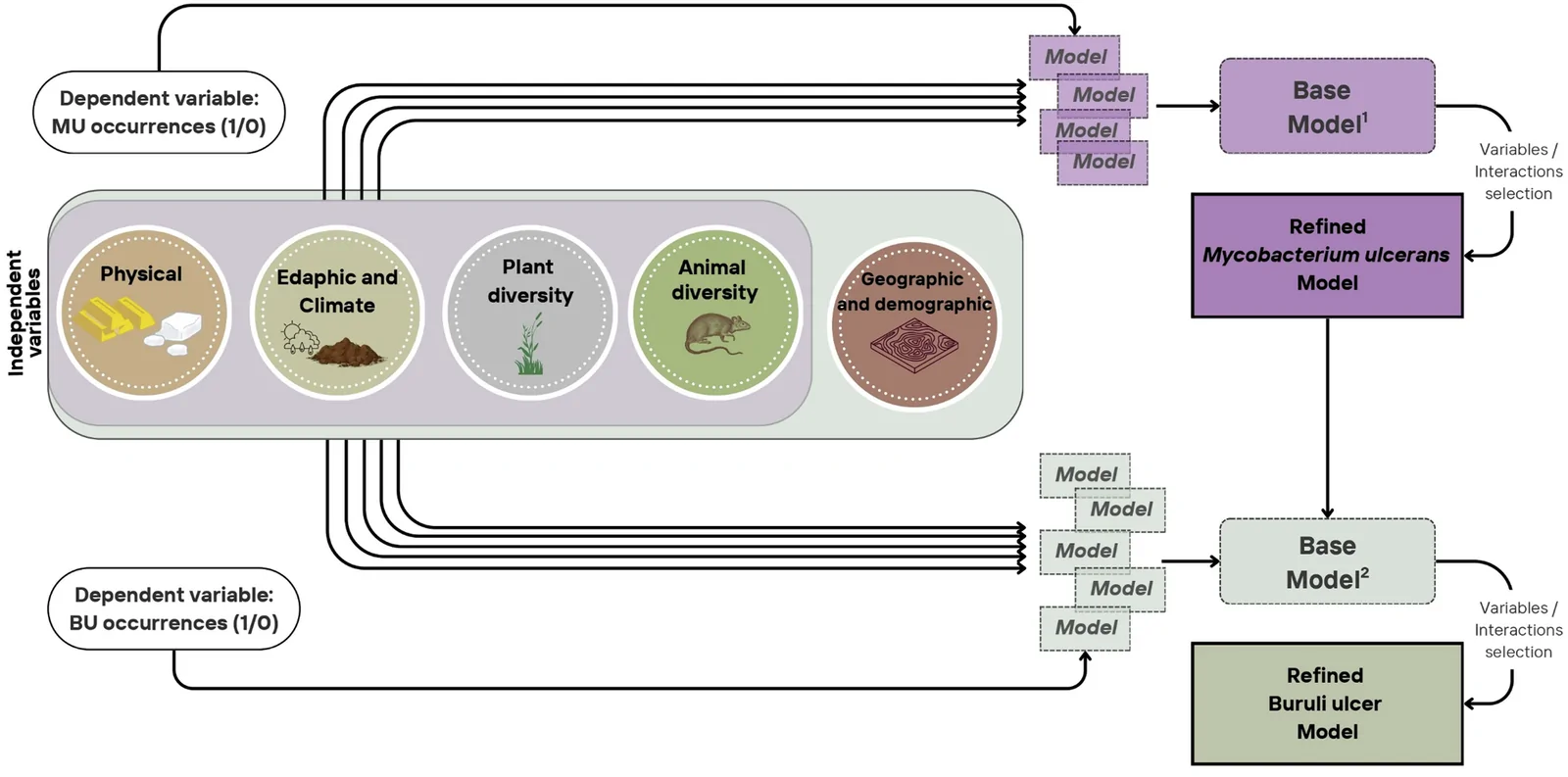
Methodological framework for *Mycobacterium ulcerans* and Buruli Ulcer modelling. For MU, four models were developed independently taking into account the variable groups and corresponding to the independent parameters. The variables selected in each of these steps were then included into a single model, resulting in the Base Model (1). A subsequent model, the RMUM, incorporated both the interactions and the variables selected from the Base Model. The *logit* equation of this latter model was used as a predictor variable for the construction of the Base Model (2) of BU. For the development of the refined Buruli Ulcer model, the same steps described above for the construction of the Refined *Mycobacterium ulcerans* Model were followed.

Our objective was to understand and map how the explanatory variables affected MU and BU distribution in Africa. For the development of generalized linear modelling (GLM), initial models were separately constructed for each parameter category. In the case of MU, four parameter categories were considered: (1) physical, (2) edaphic and climatic, (3) plant diversity and (4) animal diversity. The variables selected in this step were taken into account for the construction of a single Base Model, and subsequently a new model was developed by incorporating both the two-way interactions and the variables from the Base Model. This final model was named the refined *Mycobacterium ulcerans* model (RMUM) (Fig. 1).

For BU, in addition to the four categories listed above, a socio-demographic and geographical parameters’ group was considered. In this case, model development was completed in two stages: (1) inclusion, as a conditional predictor variable, of the RMUM *logit* equation; and (2) performing a subsequent stepwise selection to retain only those variables with potential explanatory power for BU cases. After this selection process another model was fitted, incorporating both the selected variables and their two-way interactions, resulting in the refined Buruli ulcer Model (RBUM) (Fig. 1). Variable selection was performed using IBM SPSS Statistics ver. 23 (IBM® SPSS® Statistics 2021). Although certain ecological variables, such as the presence of invasive species, may have anthropogenic origins, they were considered as biotic components. Explicit anthropogenic, social and demographic factors were incorporated in the BU model, as they modulate exposure pathways and infection risk. Distribution models were developed using the Favourability Function, previously applied in other studies on pathogens and infectious diseases^17–19^. The final set of variables retained in RMUM and RBUM are presented in the main text (Tables 1 and 2).

**Table 1:** Refined *Mycobacterium ulcerans* Model *logit* equations (i.e., linear combination of predictor variables that form part of the binomial-regression equation)

	B	S.E.	Wald	df	p
Constant	−42.191	9.34	20.406	1	0.000
Au×Soil diversity	1.008	0.229	19.313	1	0.000
Au×*Leersia hexandra*	−3.912	1.833	4.557	1	0.033
BIO-6×HNSurface	0.010	0.002	26.151	1	0.000
BIO-6×*Leersia hexandra*	0.133	0.033	15.746	1	0.000
Soil diversity×Acrisols	0.006	0.002	5.722	1	0.017
Soil diversity×*Commelina benghalensis*	0.453	0.132	11.800	1	0.001
UVB_1	0.004	0.001	9.756	1	0.002

B: variable coefficient; S.E.: ±standard error; Wald: Wald’s parameter; df: degree of freedom; p: statistical significance. Variable names are as listed in appendix pp 4-7.

**Table 2:** Refined Buruli ulcer Model *logit* equation (i.e., linear combination of predictor variables that form part of the binomial-regression equation)

	B	S.E.	Wald	df	p
Constant	4.395	0.774	32.242	1	0.000
MU_model *logit*	0.315	0.046	46.939	1	0.000
*Echinochloa pyramidalis*	1.067	0.314	11.536	1	0.001
Acrisols	0.021	0.006	11.374	1	0.001
Mn×distpop	0.000	0.000	16.874	1	0.000
Altitude×distpop	0.000	0.000	40.947	1	0.000
Altitude×Moisture	0.000	0.000	25.845	1	0.000
UVB_2×Soil diversity	0.000	0.000	20.190	1	0.000
UVB_2×Distrail	0.000	0.000	7.186	1	0.007
UVB_2×BIO-6	0.000	0.000	43.178	1	0.000
Acrisols×Moisture	0.000	0.000	5.582	1	0.018
*E. pyramidalis*×*Thryonomys swinderianus*	−1.261	0.634	3.962	1	0.047
Globc14×distpop	0.000	0.000	3.791	1	0.052
*T. swinderianus*×BIO-6	0.091	0.020	20.813	1	0.000

B: variable coefficient; S.E.: ±standard error; Wald: Wald’s parameter; df: degree of freedom; p: statistical significance. Variable names are as listed in appendix pp 4-7.

The outputs of these models were expressed as favourability scores ranging from 0 to 1, representing the degree to which natural or human conditions, in a particular spatial unit, favour the occurrence of a given event. Favourability (*F*) was calculated using the Favourability Function^20^, defined by the following formula:

$$F=\frac{\frac{P}{1-P}}{\left(\frac{{n}_{1}}{{n}_{0}}+\frac{P}{1-P}\right)}$$

*P* represents the probability of occurrence of the event under study, *n*₁ denotes the number of recorded occurrences, and *n*₀ corresponds to the number of spatial units with no recorded occurrences. The *P*-values were obtained through a forward-backward stepwise binomial regression, where predictor variables were selected based on Rao’s score tests^21^ and derived from the following equation:

$$P=\frac{{e}^{y}}{1+{e}^{y}}$$

The base of natural logarithms, *e*, and *y*, the “*logit* equation” defined as a linear combination of the selected predictor variables, were used in the model construction. Parameter estimation was carried out by iterative maximization of the log-likelihood function using a gradient ascent machine learning algorithm. The Wald’s test^22^ was employed to assess the contribution of each variable to the model.

A stepwise forward and backward selection approach was applied to avoid redundancy among predictors, allowing for the removal of predictor variables throughout the variable selection process. To further prevent excessive multicollinearity, variables with a Spearman’s correlation coefficient greater than 0.8 were not allowed within the same model. In cases where such high correlation was detected, the less significant variable was removed and the model was retained. Finally, to minimize type-I errors associated with testing many considered variables, the Benjamini-Hochberg’ procedure was applied to control the False Discovery Rate (FDR)^23^.

### Extrapolating the African BU model to tropical and subtropical regions worldwide

The RBUM, fitted to the training area in Africa, was extrapolated to worldwide tropical and subtropical regions, between latitudes 35°N and 38°S worldwide, using the following equation:

$$F=\frac{{e}^{y}}{\frac{{n}_{1}}{{n}_{0}}+{e}^{y}}$$

where *n*₁ and *n*₀ represent the number of presence and absence records, respectively, within the training area. See above for *e* and *y* definition.

Model extrapolations were restricted to hexagons where predictor variable values fell within the range of values observed in the training area. We restricted projections to a 10% tolerance range above and below to avoid extrapolation beyond the environmental space represented in the training model.

## Results

### Spatial Environmental Favourability for *M. ulcerans* in Africa

**Fig. 2:**
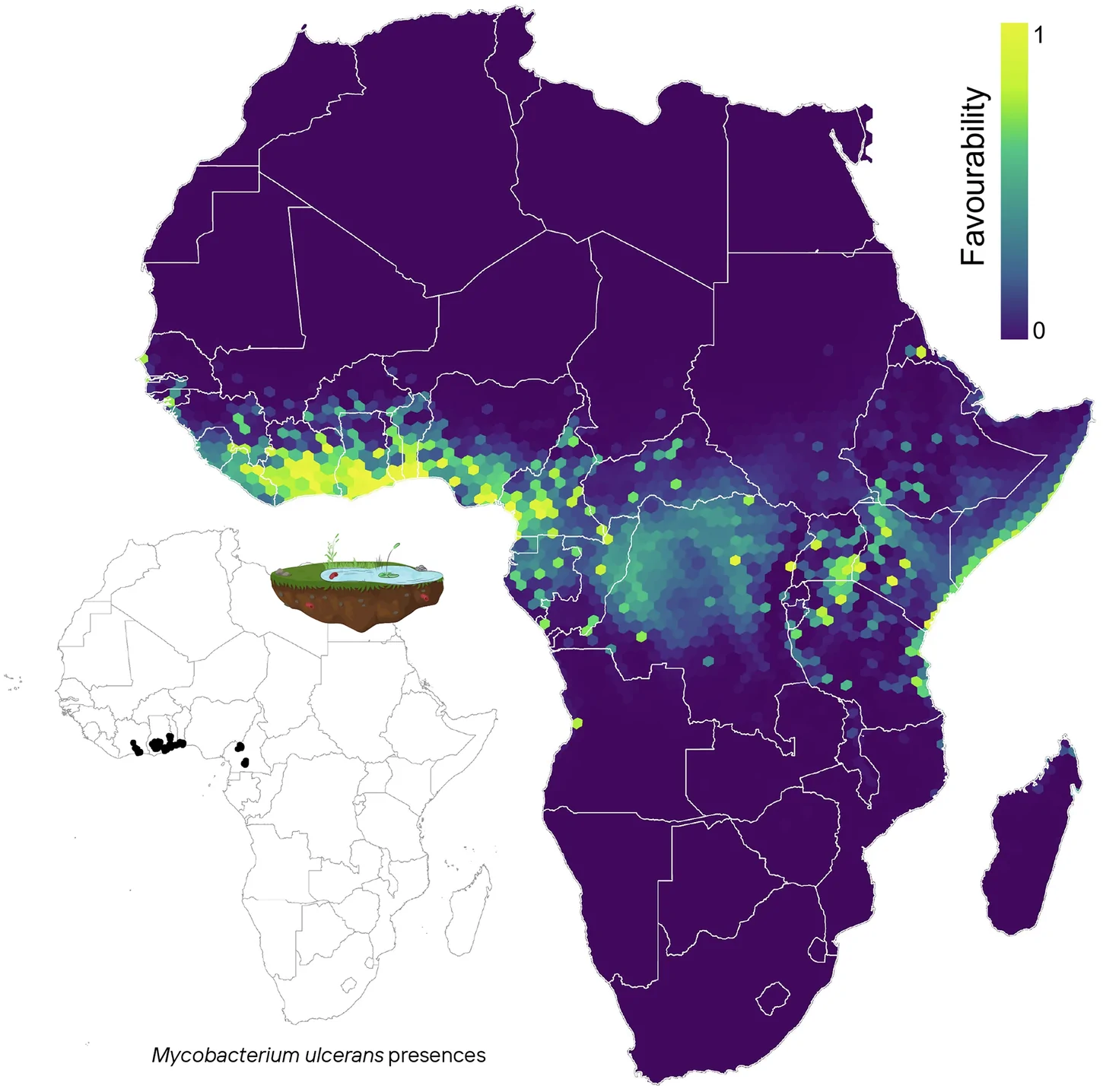
Refined *Mycobacterium ulcerans* Model (RMUM). Distribution of *M. ulcerans* occurrences (dots) observed for Africa on bottom-left panel, and predicted favourability for MU presence (illustrated in different colors) on larger panel. The maps illustrate the modeled favourability, ranging from 0 to 1, with higher values indicating areas more favourable for MU presence. The spatial resolution is based on 88×88 km^2^ hexagons.

The RMUM distribution suggests that the most favourable areas for MU are located in West and Central Africa (Fig. 2). In the Democratic Republic of the Congo (DRC), favourability was intermediate and largely concentrated in the central region of the Zaïre-Oubangui drainage area. In East Africa, favourable conditions were primarily identified in areas near Lake Victoria and along the coastal regions of the Indian Ocean. Overall, MU favourability was concentrated in areas between 10°N and 5°S (see Supplementary Information Table S1).

MU favourability areas were best explained by atmospheric and soil surface climatic parameters, one plant species distribution, gold presence and their interactions (see Table 1 for more details). These factors globally explained 51.7% of MU spatial distribution in Africa. There was a two-way interaction (partial R^2^ = 0.356, p < 0.0001) between minimal temperature of the coldest month (BIO-6) and average percent humidity near soil surface (HN-Surface) that was important to explain MU distribution (Supplementary Information Figure. S1). A positive exponential relationship was observed between MU favourability and BIO-6 for HN-surface percentage over 40%, with more favourable areas for MU presence between 15 °C and 23 °C, and 15 °C was the minimal temperature allowing MU persistence and 23 °C being the upper limit value observed (Supplementary Information Figure. S1). At 20 °C a second two-way interaction term between BIO-6 and *Leersia hexandra* contributed to explaining additional variance (partial R^2^ = 0.123, p < 0.0001) (Supplementary Information Figure. S1). Additionally, soil type diversity and gold in soil interacted to explain MU presence (partial R^2^ = 0.038, p < 0.0001), with gold presence related to more favourable MU areas (Supplementary Fig. S1).

### Spatial Environmental Favourability for Buruli ulcer cases in Africa

**Fig. 3:**
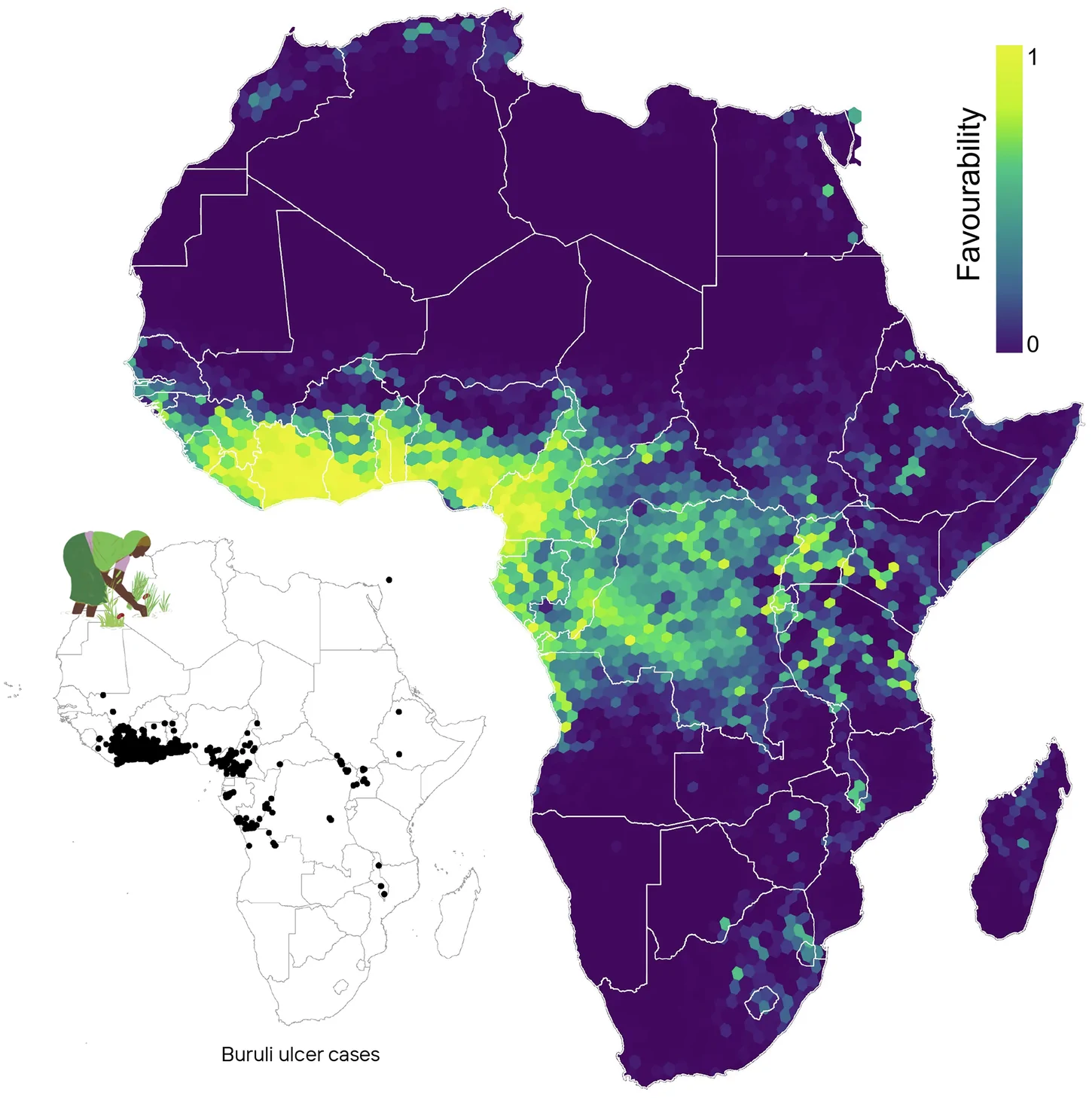
Refined Buruli ulcer Model (RBUM). Distribution of observed Buruli ulcer cases (dots) for Africa on the bottom-left panel, and predicted favourability for BU cases in the main panel. The maps illustrate the modeled favourability, ranging from 0 to 1, with higher values indicating areas more favourable for BU cases. The spatial resolution is based on 88×88 km^2^ hexagons.

Favourable areas for BU cases extend across West Africa, Central Africa, East Africa, and small regions in both Northern and Southern Africa (Fig. 3). In West Africa, the highest BU favourability was in countries such as Sierra Leone, Liberia, Côte d’Ivoire, Ghana, Benin, Togo, and Nigeria. In Central and East Africa, favourability increased relative to the environmental model for MU, particularly in Cameroon, Equatorial Guinea, the Republic of the Congo, and DRC, with additional favourable zones in Uganda, Kenya, and Tanzania. Areas of intermediate favourability were identified in North Africa (i.e., northern Algeria, the west-central region of Morocco, and several areas in Egypt) and in southern Africa (southern Malawi, northern Zimbabwe and in the humid subtropical region of South Africa) (Fig. 3). BU favourability was mainly concentrated between 10°N and 5°S, with additional zones north of 10°N (e.g., northern Niger, Senegal, Nigeria, and Egypt) and patchy areas south of 5°S in countries like Botswana, Lesotho, and Eswatini (see Supplementary Information Table S2 for more details).

BU case favourability areas were best explained by a two-way interaction between annual ultraviolet B radiation seasonality (UVB-2) and BIO-6 (partial R^2^ = 0.293, p < 0.0001) (Table 2; Supplementary Information Figure. S2), with more favourable BU areas at UVB-2 values less than 80,000 J/m²/day and BIO-6 ≥15 °C. BU favourability was also explained by MU *logit* function (partial R^2^ = 0.279, p < 0.0001) (Supplementary Information Fig. S2), and with a soil moisture by mean altitude interaction (partial R^2^ = 0.186, p < 0.0001) (Supplementary Information Fig. S2), with altitude classes ≥100 m with more pronounced soil moisture influencing BU favourability. An interaction of distance to a populated city by rainfed croplands also influenced BU favourability (partial R^2^ = 0.072, p < 0.0001) (Supplementary Information Fig. S2), indicating that the more favourable areas for BU were located near urbanization centers with rainfed cropland surfaces. Overall, these factors contributed to the total amount of 83.0% of explanation to the retained BU model.

BU presence was also explained to a lesser extent by a two-way interaction of BIO-6 and presence of *Thryonomis swinderianus* (a grasscutter rodent), (partial R^2^ = 0.068, p < 0.0001) (Supplementary Information Fig. S2). There was also effects of acrisol-type soil (i.e., rich in clay) (partial R^2^ = 0.061, p < 0.0001), the presence of the grass species *Echinochloa pyramidalis (*partial R^2^ = 0.052, p < 0.0001), an interaction of distance to a populated center and altitude (partial R^2^ = 0.032, p < 0.0001) and an interaction between UVB-2 and soil diversity (partial R^2^ = 0.031, p < 0.0001) (Supplementary Information Fig. S2). Other interactions where significant but with less than 3% of explanation to BU model (Table 2).

### Extrapolating the trained African BU model over the pantropics

**Fig. 4:**
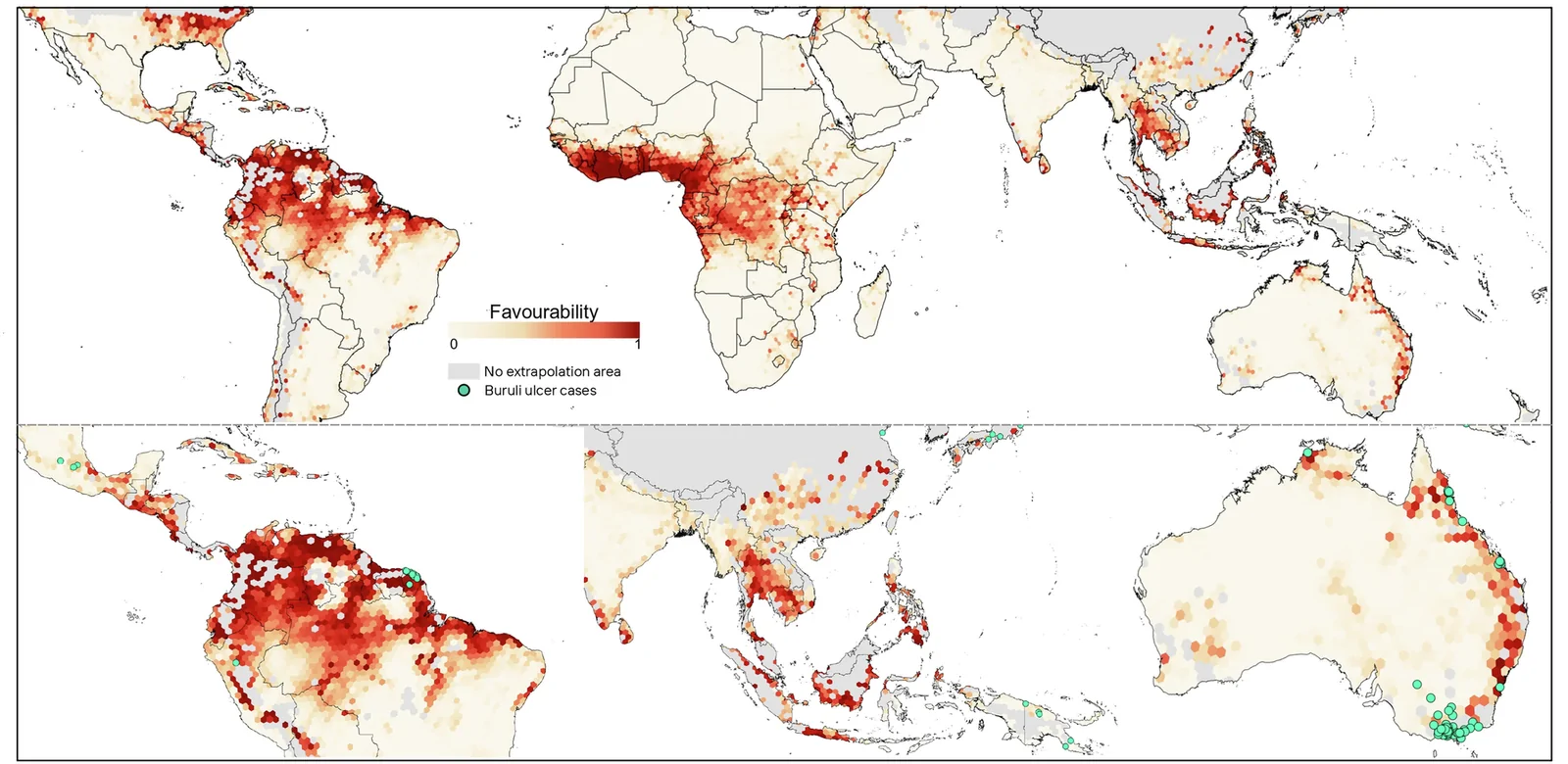
Extrapolation of the African BU model to tropical and subtropical regions worldwide. Top panel: Extrapolation of the BU model trained in Africa to global regions located between latitudes 36°N and 38°S. Bottom panel: Zoom into regions where BU cases have been reported and where fine-scale case data are available. Green dots indicate known BU case occurrences. The grey area represents regions where extrapolation is unreliable, as predictions were limited to the range of values observed in the training area. We did not fit our model for Mediterranean-like and more temperate or boreal regions, since our study included environmental, ecological conditions representative of the tropics and subtropics.

In the Americas, areas of high and intermediate BU favourability were distributed across several tropical and subtropical regions (Fig. 4). These include countries in northern South America, and parts of Central and North America, notably in regions of Mexico and the southeastern United States. In Asia, favourable zones were identified in the Indochinese Peninsula, Maritime Southeast Asia and along the northern and eastern coasts of Australia (Fig. 4). Approximately 65% of the known BU occurrence points in French Guiana, Mexico, Peru, Australia, and Japan coincided with areas predicted as highly favourable by our extrapolated model.

## Discussion

To our knowledge, this is the first effort to conduct a continental-scale spatial analyses for understanding natural and anthropogenic drivers of the spatial distribution of *Mycobacterium ulcerans* and Buruli ulcer cases. By incorporating reported occurrence data for both MU and BU and using variables not previously included in distribution modeling, our study provides high-performance models. Specifically, we emphasize: (1) the inclusion of novel important local to large-scale variables and their interaction terms with some parameters highly associated with human activities and practices that have not been jointly considered in previous studies on this disease system, (2) the pre-selection of variables within thematic parameter categories prior to their integration into a single model, and (3) the explicit integration of MU environmental favourability, i.e., including both MU and components of its ecological niche, into the extrapolated BU disease model. We discuss how to leverage interoperable databases to better understand disease patterns and processes acting at different spatial and temporal scales. By doing so, other skin NTDs, and more widely NTDs, can be studied in a similar way. Ultimately, this approach can enhance prevention and control strategies by identifying key environmental risk zones and vulnerable populations.

Any species distribution modeling has limitations in the quality of available data and interpretation of observed patterns. Poor data quality could have represented a significant constraint, particularly if the distribution of false absences were biased along environmental gradients and if the modeling techniques were prone to overfitting. Our grid lattice-based approach substantially reduced the proportion of the area classified as lacking MU and BU. Indeed, our models predict favourable conditions in 6% to 11% of hexagons initially classified as “absence” zones (see Supplementary Information Table S5 - S6). In future research, improvements to the structure and validation process of our models could be considered, facilitating their adaptation to other skin NTDs and more generally NTDs, and additional parameters of interest. Furthermore, future studies could benefit from incorporating temporal data both on MU occurrences and BU incidences carried out together in the same regions to gain a better understanding of contrasting epidemiological trends observed across regions since differential research efforts between regions could account for very contrasting current observations.

We are also conscious that assessments of model performance at the modeled spatial scales, i.e., the size of the hexagons, could lead to different results at finer scales. Our maps (see Fig. 4) illustrate the scale at which our extrapolation models are applicable (e.g., within the Orinoco River catchment basin, South America), and future work should focus on finer scale modeling.

Our spatial model provides a very good fit for the plausible distribution of MU in Africa, while allowing for an evaluation of the relative importance of both individual variables and their complex interactions. MU distribution was constrained by lower minimal temperature during the coldest month of the year (range limits between 15 °C and 23 °C, with extreme lower limit from ~ 7 to 8 °C), which prohibits its presence in more northerly or southerly latitudes, at high altitude regions, and in areas with wide temperature ranges. However, MU may be present in less favourable areas, particularly those with favourable micro-climates associated with, for example, the introduction of *L. hexandra*; an invasive plant species forming abundant mats in humid and sub-humid regions of sub-Saharan Africa, particularly in wetlands and seasonally flooded areas, which may provide suitable soil and water temperature conditions for MU establishment. This MU spatial model is limited by the few localities with reliable and confirmed presence of MU DNA in Africa^24–26^. We therefore strongly recommend considering an ecosystem-based approach to understanding such NTD diseases of sapronotic origin^27^. In contrast to MU, BU spatial distribution was based on a higher number of documented BU cases even if under-reporting is acknowledged^16^. BU appears to be represented between latitudes 11°N and 11°S, with highest likelihood across tropical West Africa, parts of Central Africa and certain regions of East Africa. This also aligns with recent finding by Simpson and colleagues^13^, who suggest suitable areas in West and Central Africa. However, in contrast, our results identify additional favourable zones in Uganda and Rwanda, several areas in South Africa and, interestingly, some areas in North Africa (i.e., the Maghreb), including Egypt, as well as fit with the findings of Vas Nunes and colleagues^28^, that suggests BU being prevalent in Sierra Leone.

Our extrapolated map (Fig. 4) clearly shows a potential quasi-pantropical distribution of BU cases. It then extends the possibility of presence to many regions of the world without reported cases, including several subtropical areas. Although MU can be present in the environment, this does not necessarily lead to cases of BU in human communities, as other physiological, immunological, genetic, socio-cultural or behavioural factors play important roles in disease transmission and progress^29^. Therefore, the BU extrapolated map should be interpreted with caution for areas where BU has yet to be reported. However, interestingly, Dogbe et al. (2025)^30^ recently reported consistent MU presence across nine aquatic habitats of the southeastern USA with no known reported cases, where our models predict MU favourability.

Our study reveals that MU is constrained by a minimum temperature that prevents its installation, with optimal conditions between 24° and 26 °C (Supplementary Information Fig. S1). HN-surface values ≥40% were also more favourable for MU (Supplementary Information Fig. S1). An interaction effect between the presence of the southern cut grass, *L. hexandra*, and BIO-6 is also observed, indicating more favourable habitats for MU in presence of this invasive grass. Our model also retained gold in soils and subsoils as more favourable to MU even if it was a weak, yet significant, association (Table 1 and Supplementary Information Fig. S1). The environmental niche of MU is most associated with temperature-associated factors and highly diverse, moist soils and subsoils potentially containing high levels of gold. To our knowledge these are the first models demonstrating MU spatial distribution consistently correlated with environmental, ecological conditions.

Previous research has shown seasonal temperature variation is important to bacterial taxa diversity in soils and freshwaters^31^, with bacterial communities of hosts associated with bioclimatic conditions at the global scale^32^. We confirm these findings for MU at the African continental scale. It is also accepted that soil enzymatic and microbial activity tends to increase with higher soil moisture content^33^, which can provide explanations for the importance of soil water balances in the ecological niches of MU and other mycobacterial pathogens. The positive effect of the invasive pantropical grass species, *L. hexandra*, on MU environmental distribution is consistent with soil-water balance. This plant grows from rhizomes and stolons in aquatic or semi-aquatic ecosystems, and the stems can become matted in aquatic habitats forming carpets that may benefit MU persistence below in normally adverse microhabitats. *L. hexandra* is known for its tolerance and hyperaccumulation of diverse metals (e.g., copper, nickel, zinc)^34,35^ and displayed a significant interaction with gold in soils in our models. Our continental-scale investigation corroborates previous investigations associating gold or gold mining activities with increased BU risk^36^; however, the extent to which this is due to direct impacts of gold (or other metals involved in gold mining, such as arsenic or mercury) *versus* mining activities that expose miners to MU contaminated soil or water remains unknown^36,37^.

Approximately 29.3% of the variation in the BU model was explained by sunlight radiation seasonality (UVB-2) interacting with climatic and edaphic conditions. Another 27.9% of the variation in BU distribution relies, on the MU *logit* values. In addition, cropland near cities was important in models of BU case distribution. Previous studies have identified peri-urban areas, extensive agriculture, and mining activities associated with BU cases^38^. Additional but less influential factors (accounting for 6% of BU cases overall) included the interaction between BIO-6 and the rodent *Thryonomys swinderianus*. Although disease caused by MU has been described in domestic animals in Africa^39^, it could also affect wild animals, as suggested by Hammoudi et al. (2020)^40^ and Durnez et al. (2010)^41^. On the other hand, this grass-cutter rodent could act as a host disperser for MU through feces. Minor contributions to the spatial distribution of BU cases (about 5-6% as a subtotal) was also observed from the presences of acrisols, i.e., clay-rich soil types, and the grass species, *Echinochloa pyramidalis*.

Ultraviolet B (UV-B) radiation has potentially severe ecological and human health impacts, especially with other stressors such as extreme temperatures^42^. Our models concur with previous research showing MU to be highly susceptible to UV irradiation, with temperature mediating its metabolic responses^43^, and additional experiments highlighting UV radiation as a relevant factor for explaining BU spatial distribution in Africa^44^.

Interestingly, two invasive grass species had significant effects in our GLM models: the interaction between BIO-6 and the presence of *L. hexandra* (explaining 12% of MU favourability) while the presence of *E. pyramidalis* explained an additional 5% for BU. These grasses, which benefit from rhizomes that expand their local distribution, share high tolerance to seasonal drought and flooding^45,46^, and to human-induced pollution, including oil and heavy metals^47,48^. They also constitute forage resources for livestock^49^, thus have benefited from long distance dispersal associated with anthropogenic activities^50^. As a grass, *E. pyramidalis* may also contribute to MU transmission to human via skin trauma^51^.

There is still considerable controversy as to whether MU has a reservoir host and insect-borne transmission (but see discussions by Chevillon and colleagues^11^ and Guégan and colleagues^27^. In the absence of a known reservoir or vector in Africa^25,52^, it is very interesting that our models indicated an interaction between BIO-6 and the presence of a rodent, *T. swinderianus*, that has a small, but significant effect, on BU spatial distribution. As an obligate herbivore of leaves, roots and cereals, this rodent is sometimes reared as micro-livestock and is a pest in sugar cane, wheat and maize fields^53^. Throughout Africa, this rodent is abundant in wetlands and semi-humid areas, especially those densely covered by grasses^53^. Such landscapes thus combine characteristics previously suggested as favouring both MU environmental persistence (wetlands with vegetal cover) and MU contacts with human derma (grass leaves cutting skin).

In conclusion, our findings represent a significant advance in understanding a category of NTDs whose causative agent is recognized as having an environmental origin, i.e., sapronotic agent. Our work offers an approach integrating physical, bioclimatic, environmental, ecological, socio-demographic and geographical data to better understand the distribution of *M. ulcerans* and Buruli ulcer. This involves establishing coupled environmental and health surveillance systems and adopting an approach that considers such pathogens to be more widely distributed in the environment, with disease cases emerging under specific and multifactorial, interrelationships between several parameters conditions. However, the modeled BU distribution that we present should be treated with caution, as additional consideration of complex interactions of human exposure and sociocultural conditions also likely play a role in pathogen occurrence, persistence, spread and transmission. Expanding this type of research to other cutaneous infectious and parasitic diseases, and more generally NTDs for which the disease agent is embedded into natural ecosystems, will help advance global public health by accounting for global environmental changes.

Furthermore, the ecology and epidemiology of these diseases are undergoing transformation, influenced by drivers such as the spread of invasive plant species, land-use change, and agricultural development notably close to large cities, i.e., in ultra-peripherical areas of large cities, all of which can significantly alter disease system dynamics. These findings highlight the need for close collaboration between environmental and ecological scientists, and epidemiologists and public health specialists to effectively address the growing impact of neglected (cutaneous) diseases in the context of global environmental and social changes.

### Statement on ethics approval

This study is a secondary analysis based entirely on publicly available, de-identified epidemiological and mapping data extractsed from peer-reviewed literature; no primary human participants and no patient clinical charts were accessed or managed by the authors. Consequently, institutional review board (IRB) review and primary informed consent were waived for this specific analysis. The ethical approvals, IRBs, and regulatory frameworks governing the primary data collection of each source publication are detailed below:

- Brou et al. (2008) & Bayonne et al. (2013): Formally conducted under the epidemiological surveillance mandates and public health data-reporting guidelines approved by the National Program for Buruli Ulcer Control from the Ministry of Public Health of Côte d’Ivoire and Gabon, respectively. Data collection for Bayonne et al. (2013), was granted by the World Health Organization.
- Marion et al. (2014): Formally approved by the National Ethics Committee for Health Research and executed in direct collaboration with the National Leprosy, Buruli Ulcer, and Yaws Control Program.
- Vandelannoote et al. (2017): Permission for the study was obtained from the ITM Institutional Review Board.
- Pileggi et al. (2017): Approved by the National Ethics Committee for Health Research and the National Buruli Ulcer Control Programme.
- Simpson et al. (2019) & Hammoudi et al. (2020): Utilized historical, aggregated open-access data and public health consensus repositories. Primary individual informed consent and specific IRB review were waived under national public health surveillance exemptions and WHO data-reporting frameworks.

This study and data used were approved according to French national frameworks coordinating spatial mapping protocols, and in compliance with the Declaration of Helsinki.

## Supplementary information

Transparent Peer Review file

Supplementary Information

## Data Availability

Datasets with the occurrence of *Mycobacterium ulcerans* in water and soil and reported human cases of Buruli ulcer in a hexagonal spatial grid in Africa, along with the sources of this information, are available in the Figshare repository https://doi.org/10.6084/m9.figshare.31224184^54^.
