# Supplementary material for: Human-induced pressures and environmental factors drive the distribution of neglected tropical disease Buruli ulcer
Source: Commun Med (Lond). 2026 Jun 4;6:536. doi: 10.1038/s43856-026-01692-6 (PMC13672292; doi:10.1038/s43856-026-01692-6)
Supplement: Supplementary file 1 [file 43856_2026_1692_MOESM1_ESM.pdf]

nature portfolio

Peer Review File

Human-induced pressures and environmental factors drive the distribution of neglected tropical disease Buruli ulcer

Corresponding Author: Dr Alisa Aliaga-Samanez

This file contains all reviewer reports in order by version, followed by all author rebuttals in order by version.

Version 0:

Reviewer comments:

Reviewer #1

(Remarks to the Author)
The manuscript by Dr. Aliaga-Samanez and colleagues proposes a disease ecology approach to identify human-induced pressures and environmental factors driving the spatial distribution of Buruli ulcer, a skin neglected tropical disease caused by the bacterial pathogen Mycobacterium ulcerans. The authors make use of generalized linear models to describe the distribution of the causative agent and the occurrence of human Buruli ulcer cases. The distribution models showed that M. ulcerans favourability is tied to specific warm, moist environmental niches, whereas the distribution of Buruli ulcer cases is largely modulated by low seasonal UV radiation and human activity near rainfed croplands and cities, suggesting the potential for a quasi-pantropical distribution. The topic of the manuscript is quite interesting and certainly well-suited for this journal. The analysis seems to be conducted in a robust way, and the results seem sound. The manuscript is also well written and organized. All this to say that I have only relatively minor comments, detailed below:

- l.99: the word "relatively" is key here and would need better framing to become actually informative. I would suggest to move this comment on the spatial resolution to the Methods section, where it can be contextualized (i.e., put in the context of other similar studies);
- l.117: the description of the most influential variables for the distribution of M. ulcerans should be improved. For instance, "one plant species" is not informative enough: which one? Also, "and their interactions": between which pair of variables?
- l.122: what kind of test has been performed to show that the relationship between the minimum temperature of the coldest month and M. ulcerans favorability is indeed "exponential"?
- l.185: I would suggest treating this extrapolation exercise with a grain of salt. I think that the most critical element, here, is that the extrapolation does not refer to the ecological niche of a single species – as often done in habitat suitability modeling. Here, the variable being extrapolated is the reported incidence of Buruli ulcer cases. I think it could be useful to think about the transmission process in terms of risk evaluation. While the hazard component (the distribution of M. ulcerans) could be reasonably learned from the geographical regions where the observation data comes from and applied elsewhere, the exposure and vulnerability components might prove trickier to extrapolate from the original context. Human behavior (which strongly influences exposure) and the health-care infrastructure (which strongly influences vulnerability and reporting rates) might greatly vary from one country to another. At the very least I would suggest stressing that what is being modeled is actually a potential distribution of Buruli ulcer cases – but a more careful framing of this exercise seems to be warranted;
- l.199: I wonder whether there exist better methods to assess the reliability of this extrapolation without introducing such a strict threshold. I think that the literature on uncertainty analysis applied to species distribution models could be a good starting point;
- l.209: it is unclear whether the model performances have been tested only during training or also on validation datasets. In the former case, the reported performances might greatly overestimate the actual extrapolation skills of the models. In the latter, it would be important to give some details about how the data was split between training and validation datasets;
- l.212: I agree that the interpretation of spatial patterns produced by species distribution models may not be straightforward, but at least generalized linear models should be highly interpretable. In addition, many tools have been proposed over time to `open the black box' for less interpretable algorithms. I think that some of these tools could be usefully applied also for the problem at hand. For instance, Shapley values are model-agnostic and may offer a unified and consistent framework for model interpretation;
- l.242: I would stress once again that the distribution should be intended as potential

Typos and other comments

- l.118: a sentence cannot start with “Which”
- l.124: 23°C; -> 23°C,
- l.171: grass species, Echinochloa pyramidalis, -> grass species Echinochloa pyramidalis
- l.211: “limitations of uncertainties quality” is quite unclear
- l.215: approach, -> approach
- l.246: behavior -> behavioral
- l.252: of between -> between
- l.253: observed -> observed,
- l.313: mostly -> is mostly
- l.361: “inclusion… RMUM” unclear
- l.406: this step -> these steps

Reviewer #2

(Remarks to the Author)
Title and Introduction:
Based on the title, the objective of this manuscript is to identify environmental factors and human-induced pressures to study the spatial distribution of a cutaneous neglected tropical disease. Since the focus here is Buruli ulcer, it would be important to mention it in the title, as skin NTDs encompass more than a dozen diseases, all very different from one another.
It is problematic to present and discuss skin NTDs by citing Buruli ulcer, leprosy, yaws, and cutaneous leishmaniasis in the first lines of the introduction, since these diseases are caused by completely different microorganisms, with environmental or non-environmental origins, transmissible or not between humans, and requiring or not requiring a vector in their life cycle.
We understand later that the manuscript focuses on M. ulcerans, an environmentally derived pathogen. Among the other skin NTDs, some also have an environmental origin (mycetoma, chromoblastomycosis), but this is not mentioned in the introduction and no later either.
It is therefore difficult to understand the originality of this study relative to the current state of knowledge. Indeed, reference number 13 cited at the end of the introduction is titled “Mapping suitability for Buruli ulcer at fine spatial scales across Africa: A modelling study.” How do the authors position themselves in relation to this study? The maps produced in the manuscript strongly resemble those presented in this 2021 publication.

Methods:
Data source:
For a study of this kind, relying on diverse data involving the presence of a pathogen in the environment and/or the detection of diagnosed human cases, one would expect this section to be detailed. Instead, it refers to two supplemental tables, which themselves refer to multiple references that must then be retrieved. To understand the origin and selection of the data, one essentially must reread six additional articles. The main source of the data comes from a recent article by Simpson et al. (2019), “Mapping the global distribution of Buruli ulcer: a systematic review with evidence consensus.”
Pathogeographical approach:
We understand that this is the core of the analysis and the manuscript. However, for a reader who is not a modelling specialist, it is difficult to link this methods section with the two tables presented in the results and the variables listed in them.
Results section:
The first sentences of the first and second paragraphs state:
“The RMUM distribution suggests that the most favourable areas are located in West and Central Africa (Figure 1).”
“Favourable areas for BU cases extend across West Africa, Central Africa, East Africa, and some small regions in both Northern and Southern Africa (Figure 2).”
A modelling effort is not needed to know where Buruli ulcer occurs and where M. ulcerans is detected. Furthermore, this is not the first modelling study to produce maps of this precision. It is difficult to understand how the authors position their work relative to the existing literature.
The results then present associations with different variables, which are discussed later, such as the presence of L. hexandra, a plant species, or T. swinderianus, a rodent. It would have been important to present information on their geographic ranges and their dynamics within the studied regions.
Discussion:
Again, this statement is problematic due to the variability among skin NTDs:
“In conclusion, our findings represent a significant advance in understanding skin NTDs by offering an approach integrating physical, bioclimatic, environmental, ecological, socio-demographic and geographical data to better understand the distribution of M. ulcerans and Buruli ulcer.”
The limitations of the modelling are appropriately noted, particularly the uncertainty, the quality of available data, and the interpretation of observed patterns. In the context of Buruli ulcer control, some African countries are more advanced than others—for example in active case detection with systematic qPCR confirmation, or in environmental detection of M. ulcerans. Would it not be possible to validate the model results at a finer scale by selecting study areas where more reliable data are available?
Finally, the manuscript does not address the dynamics of Buruli ulcer detection rates in Africa, which are declining, whereas detection rates in Australia continue to rise. How could the authors’ results explain such contrasting trends?

Reviewer #3

(Remarks to the Author)

What are the major claims of the paper?
The authors claim that “this is the first effort to use high-resolution, continental scale spatial analyses for understanding natural and anthropogenic drivers of MU and BU distribution”.
This claim is not supported as the analysis is at a coarse resolution, does not include spatial effects, does not truly identify “drivers” of either outcome, and does not adequately acknowledge earlier efforts to predict suitability for them.
Fine scale, continental level environmental suitability models for M. ulcerans and Buruli ulcer were published by Simpson et al in 20211. This work is briefly mentioned in the introduction section of this manuscript but not in the discussion, which seems like a missed opportunity to compare and contrast findings.
This study also appears to rely heavily on the datasets generated through the earlier study, although the datasets themselves are not cited.
This study may improve upon the earlier study in some ways: mostly by using a more ecologically grounded approach to variable selection. However, many aspects of this work are described as though they are original, which overlooks the contribution of the earlier study.
Examples:
1) “Limited work has been done on the regional and continental distribution of MU and BU, and when such studies do exist, they mostly estimate environmental suitability without identifying its main drivers.”
In fact, the Simpson et al study did quantify associations of environmental covariates with suitability for BU and MU using a comparable (though not identical) approach, though these associations were not interpreted to “drive” suitability. The current study does interpret correlations in this way, but without the necessary support to infer causality (or "driving"). It’s not clear how the authors are justifying their claims that environmental correlates of favourability represent drivers rather than indicators of suitability.
2) “To our knowledge these are the first models demonstrating MU spatial distribution consistently correlated with environmental macro-environment conditions”
It’s not clear what the authors are aiming to say here. I interpret this to mean that correlations between favourability score and macro-environmental covariates are consistent across space, but there is no evidence from results shown in the manuscript to support this. In fact the scatterplots in Fig S1. seem to contradict this: correlations between favourability scores and environmental covariates vary between levels of other covariates.
The Simpson et al study also showed associations of MU with macro-environmental covariates, and the overlap in the factors identified would be interesting to elaborate on in the discussion.
The authors further state: “Using a relatively fine spatial scale of resolution, 88×88 km2 and an extensive dataset of independent parameters, we show how natural and anthropogenic factors influence the current geographic ranges of MU and BU cases.”
The resolution of the Simpson et al study was 5x5km; i.e. almost 300x higher than in this study, so the claim that this is “relatively fine” is not supported. A resolution of 88km2 is unsuitable for modelling the distribution of such a focal disease. Its extreme localisation within endemic countries suggests that ecological drivers of MU & BU operate on a much finer scale via local interactions and effects. Evidence from several sources is summarised in the introduction of Landier et al 2.
Presumably due mostly to the coarse resolution used, predicted favourability for BU is very broad (covering most of the areas represented by occurrence records) and I question the public health utility of the finding that all regions of 6 countries are highly favourable for the disease. Given that BU is highly focal, with low and declining incidence, how will these findings support its control?
I believe that the coarse resolution of analysis is the main flaw in this study

Abstract
The abstract is not scientific; it overstates the significance of the work and does not describe the methods or the key results.
Discussion
False claims: “Although disease caused by MU has never been described in wild or domestic animals in Africa, this may reflect a lack of attention to the presence of the disease in animal hosts in general” MU has been identified in clinical samples from lesions of several domestic animals in Benin 3
Vague conclusions: “Expanding this type of research to other cutaneous infectious and parasitic diseases will help advance global public health by accounting for global environmental changes” What type of research are you advocating for? Which infections/ diseases? How can this account for global environmental changes?
Further issues
Supplementary:
More information is needed about “R2-partial” values; I assume these should be described as partial R2
Figure legends should be wholly self-explanatory, and should not state “See main text for further explanation”. What is HN-Surface?

Appropriateness and validity of any statistical analysis
There is no information about how absences were identified. If simply absence of occurrence, they should consider how under detection may lead impact findings.
The authors appear to have indiscriminately allowed for interactions between selected variables (p13 lines 363- 365): “After this selection process another model was fitted, incorporating both the selected variables and their two-way interactions, resulting in the refined Buruli ulcer Model (RBUM)”. This tolerance for interaction terms has led to dominance of drivers which are very difficult to interpret: e.g. “approximately 29.3% of the variation in the BU model was explained by sunlight radiation seasonality (UVB 2) interacting with climatic and edaphic conditions… Additional but less influential factors (accounting for 6% of BU cases 284 overall) included the interaction between BIO-6 and the rodent Thryonomys swinderianus.”
It is generally recommended that interaction terms should be allowed only when there is prior evidence or theoretical support for a non-additive relationship.

Reproducibility
Possible, although neither study datasets nor code are provided.

1 Simpson, Hope, et al. "Mapping suitability for Buruli ulcer at fine spatial scales across Africa: a modelling study." PLoS neglected tropical diseases 15.3 (2021): e0009157.
2 Landier, Jordi, et al. "Spatio-temporal patterns and landscape-associated risk of Buruli ulcer in Akonolinga, Cameroon." PLoS neglected tropical diseases 8.9 (2014): e3123.
3 Djouaka, Rousseau, et al. "Domestic animals infected with Mycobacterium ulcerans—Implications for transmission to humans." PLoS neglected tropical diseases 12.7 (2018): e0006572.

Reviewer #4

(Remarks to the Author)
I co-reviewed this manuscript with one of the reviewers who provided the listed reports. This is part of the Communications Medicine initiative to facilitate training in peer review and to provide appropriate recognition for Early Career Researchers who co-review manuscripts.

Version 1:

Reviewer comments:

Reviewer #1

(Remarks to the Author)
I have now reviewed the revised manuscript by Dr. Aliaga-Samanez and colleagues. I believe the authors have done a good job of addressing my comments and updating the manuscript accordingly. In particular, I appreciated the clarifications regarding the model's validation and the interpretability of the results.

However, I am less convinced by the authors' response regarding the extrapolation of the BU model to pantropical regions outside those used for training in Africa. Perhaps my comments were not entirely clear in the previous review stage (apologies if that was the case), but I feel the authors have conflated two distinct points in their response.

The first point referred to the reportedly exponential relationship between temperature and pathogen favorability (line 215 in the revised manuscript). While I agree with the authors that, in this context, an exponential function may not be the most biologically interpretable (plus, the forced capping at one may not be the most elegant solution in purely mathematical terms), if it fits the data, that is fine with me.

The second point referred to the section titled 'Extrapolating the African BU model to tropical and subtropical regions worldwide' (line 266 in the revised manuscript). It is clear from this section that the authors are using their RBUM, which is an explanatory model for reported cases. I would not have made this point if the authors had used their RMUM for extrapolation; once uncertainty is accounted for, this is a standard exercise in habitat distribution modelling. The problem with the RBUM is that case distribution depends on several context-dependent socioeconomic factors (the exposure and vulnerability components of the risk equation that I mentioned) that cannot simply be treated in the same way as environmental or climatic variables. This is why I suggested interpreting the results shown in Figure 4 with some caution.

Reviewer #2

(Remarks to the Author)
All my previous comments regarding the introduction have not been adequately addressed, despite the statement in the point-by-point responses: “Thank you for your comments, which have substantially improved the manuscript.” The main issue remains that the current version does not clearly present the state of the art or the specific added value of this manuscript compared with previous studies.

Furthermore, the sentences at the end of the introduction — “We discuss how to leverage interoperable databases to better understand disease patterns and processes acting at different spatial and temporal scales. By doing so, other skin NTDs, and more widely NTDs, can be studied in a similar way. Ultimately, this approach can enhance prevention and control strategies by identifying key environmental risk zones and vulnerable populations” — do not seem appropriate for this section. These statements are highly speculative and would be more suitable for the discussion.

Regarding the comment raised during the review process:

“Finally, the manuscript does not address the dynamics of Buruli ulcer detection rates in Africa, which are declining, whereas detection rates in Australia continue to rise. How could the authors’ results explain such contrasting trends?”

The response provided is not satisfactory. It is difficult to claim that a model identifies factors likely to explain the presence of MU/BU without incorporating temporality and explicitly addressing the epidemiological dynamics of the disease. Ignoring these temporal trends considerably limits the explanatory power and relevance of the proposed model.

Reviewer #3

(Remarks to the Author)
The authors have mostly addressed the key points raised though there are two outstanding issues.
1. I agree with the Reviewer 2 comment that Buruli ulcer should be included in the title but this change has not been made.
2. The authors have soundly explained their reasoning for the use of an 88km2 grid. However the first line of the discussion still states “To our knowledge, this is the first effort to use high-resolution, continental scale spatial 278 analyses for understanding natural and anthropogenic drivers of the spatial 279 distribution of Mycobacterium ulcerans and Buruli ulcer cases.” I do not think that any experienced reader would consider the scale of the models “high-resolution”. I suggest they drop this descriptor.

Version 2:

Reviewer comments:

Reviewer #1

(Remarks to the Author)
The authors have clarified the only remaining issue I raised in the previous review round. I appreciate their response and the clarification they have included in the revised manuscript. I have no further comments on this new version and congratulate the authors on their interesting work.

Reviewer #2

(Remarks to the Author)
Compared to the results described in the manuscript (fewer than 800 words), the discussion appears disproportionate (nearly 2,000 words) and somewhat unfocused, which limits understanding of the manuscript’s impact. The discussion should focus on placing the results in perspective with respect to the existing literature.

Open Access This Peer Review File is licensed under a Creative Commons Attribution 4.0 International License, which permits use, sharing, adaptation, distribution and reproduction in any medium or format, as long as you give appropriate credit to the original author(s) and the source, provide a link to the Creative Commons license, and indicate if changes were made.
In cases where reviewers are anonymous, credit should be given to 'Anonymous Referee' and the source.
The images or other third party material in this Peer Review File are included in the article’s Creative Commons license, unless indicated otherwise in a credit line to the material. If material is not included in the article’s Creative Commons license and your intended use is not permitted by statutory regulation or exceeds the permitted use, you will need to obtain permission directly from the copyright holder.
To view a copy of this license, visit https://creativecommons.org/licenses/by/4.0/

Manuscript COMMSMED-25-2667-T

Reviewers' comments:

Reviewer #1 (Remarks to the Author):

The manuscript by Dr. Aliaga-Samanez and colleagues proposes a disease ecology approach to identify human-induced pressures and environmental factors driving the spatial distribution of Buruli ulcer, a skin neglected tropical disease caused by the bacterial pathogen Mycobacterium ulcerans. The authors make use of generalized linear models to describe the distribution of the causative agent and the occurrence of human Buruli ulcer cases. The distribution models showed that M. ulcerans favourability is tied to specific warm, moist environmental niches, whereas the distribution of Buruli ulcer cases is largely modulated by low seasonal UV radiation and human activity near rainfed croplands and cities, suggesting the potential for a quasi-pantropical distribution. The topic of the manuscript is quite interesting and certainly well-suited for this journal. The analysis seems to be conducted in a robust way, and the results seem sound. The manuscript is also well written and organized. All this to say that I have only relatively minor comments, detailed below:

Reply. Thank you for your positive remarks and thorough review, which greatly improved our work.

- l.99: the word "relatively" is key here and would need better framing to become actually informative. I would suggest to move this comment on the spatial resolution to the Methods section, where it can be contextualized (i.e., put in the context of other similar studies)

Reply. It has been done.

- l.117: the description of the most influential variables for the distribution of *M. ulcerans* should be improved. For instance, "one plant species" is not informative enough: which one? Also, "and their interactions": between which pair of variables?

Reply. We thank the reviewer for this comment, and we improved the description of the most influential variables in this new version of our manuscript. Concerning the interactions and since there are several interactions, we decided it was best to show them in Table 1 itself; we have included "see Table 1 for more details " in line 211.

- l.122: what kind of test has been performed to show that the relationship between the minimum temperature of the coldest month and *M. ulcerans* favorability is indeed "exponential"?- l.185: I would suggest treating this extrapolation exercise with a grain of salt. I think that the most critical element, here, is that the extrapolation does not refer to the ecological niche of a single species – as often done in habitat suitability modeling. Here, the

variable being extrapolated is the reported incidence of Buruli ulcer cases. I think it could be useful to think about the transmission process in terms of risk evaluation. While the hazard component (the distribution of M. ulcerans) could be reasonably learned from the geographical regions where the observation data comes from and applied elsewhere, the exposure and vulnerability components might prove trickier to extrapolate from the original context. Human behavior (which strongly influences exposure) and the health-care infrastructure (which strongly influences vulnerability and reporting rates) might greatly vary from one country to another. At the very least I would suggest stressing that what is being modeled is actually a potential distribution of Buruli ulcer cases – but a more careful framing of this exercise seems to be warranted;

Reply. We are grateful to the reviewer for these important comments, and we agree with most of them, notably concerning the difference between hazard(s) and risk(s). First, concerning the curve shape of minimum temperature of the coldest month and *M. ulcerans* favourability in interaction with HN-Surface categories, being "exponential" (please see Supplementary Information, Fig. S1a), we tested different curve shapes, notably expecting a *S*-shape trend with a plateau. This latter function would have been biologically easier to interpret, highlighting the existence of a temperature threshold beyond which favourability would decrease; however, this is not visible from the data. The exponential trend of the current curve shows an increase in MU favourability for values of minimum temperature of the coldest month above 20°C, and for HN-Surface categories between 40 and 50 and >50 (but not for those below 40). It is not BU favourability that is extrapolated (see part of the sentence above: "the variable being extrapolated is the reported incidence of Buruli ulcer cases"), but MU that is extrapolated in generating MU favourability areas. It therefore appears that MU, or rather MU favourability, is highly dependent on temperature limits, with excessively cold temperatures during the coldest month being associated with lower persistence and development of MU, and 23°C (optimal range 20°C-23°C) being the limit value in the coldest month for persistence and development of the bacilli.

We agree with the reviewer's point about hazard(s), exposure(s), vulnerability(ies) and infectious risk, and we understand what infectious risk is and the complexity of calculating and assessing it. However, BU is not a contagious disease, and therefore a point of presence for MU susceptibility when there is exposure may or may not result in a case of illness, depending on vulnerability(ies). Interestingly, the number of cases in the state of Victoria in Australia shows that people are more exposed to environments favourable to MU than they are vulnerable, as these populations are largely of European origin and are known to be more resistant to MU infection. According to this and the fact that we did not take into consideration human behaviours and attitudes, and individual and community vulnerabilities, we developed potential distribution of Buruli ulcer cases as recommended by the reviewer, requesting for a more careful framing the present work.

- l.199: I wonder whether there exist better methods to assess the reliability of this extrapolation without introducing such a strict threshold. I think that the literature on uncertainty analysis applied to species distribution models could be a good starting point;

Reply. We only accepted a 10% tolerance. This precaution avoided projections into areas that were not environmentally represented in the region used for model training (e.g., Farfán et al. 2019). We included this information in the main text (lines 195-198).

Farfán, M. A., Aliaga-Samanez, A., Olivero, J., Williams, D., Dupain, J., Guian, Z., & Fa, J. E. (2019). Spatial modelling for predicting potential wildlife distributions and human impacts in the Dja Forest Reserve, Cameroon. Biological Conservation, 230, 104-112.

- l.209: it is unclear whether the model performances have been tested only during training or also on validation datasets. In the former case, the reported performances might greatly overestimate the actual extrapolation skills of the models. In the latter, it would be important to give some details about how the data was split between training and validation datasets;

Reply. Due to the limited number of presence records for *M. ulcerans* and Buruli ulcer, we decided to use all available presences and not reserve a portion for model validation. However, in the case of the *M. ulcerans* model, we validated it using Buruli ulcer case occurrences. The model performed well both on the data used for training and on the independent disease occurrence data used for validation. This validation is included in the new Table S6 of the Supplementary Information file in the revised version.

- l.212: I agree that the interpretation of spatial patterns produced by species distribution models may not be straightforward, but at least generalized linear models should be highly interpretable. In addition, many tools have been proposed over time to `open the black box’ for less interpretable algorithms. I think that some of these tools could be usefully applied also for the problem at hand. For instance, Shapley values are model-agnostic and may offer a unified and consistent framework for model interpretation.

Reply. We thank the reviewer for this interpretation and for their advice to use Shapley's values to interpret the results of our statistical models. Some of us have used almost identical procedures. However, given that we have introduced interactions into our statistical models (we are also among the first to do so in this type of analysis), we would like to emphasize that in most situations, it is these two-way interaction terms that explain the variables we want to explain (MU and BU favourability, respectively). In other words, these are complex products of variables that explain the observations, and we preferred not to introduce three-way interactions because they are difficult to explain from a biological and epidemiological point of view. So, the main message is that it is multifactorial conditions involving interrelated variables (rather than single factors) that account for our observations, clearly indicating the inherent complexity of this type of infectious system. This is also why using this same type of analysis should reveal the complexity of other infectious systems.

- l.242: I would stress once again that the distribution should be intended as potential.

Reply. We agree. We have included "potential" in line 329.

Typos and other comments

- l.118: a sentence cannot start with “Which”

Reply. Done. We have replaced “Which” with “These factors”.

- l.124: 23°C; -> 23°C,

Reply. Done

- l.171: grass species, Echinochloa pyramidalis, -> grass species Echinochloa pyramidalis

Reply. Done

- l.211: “limitations of uncertainties quality” is quite unclear

Reply. We have corrected the sentence.

- l.215: approach, -> approach

Reply. Done

- l.246: behavior -> behavioral

Reply. Done

- l.252: of between -> between

Reply. Done

- l.253: observed -> observed,

Reply. Done

- l.313: mostly -> is mostly

Reply. Done

- l.361: “inclusion… RMUM” unclear

Reply. Done. We have modified the sentence to make it clearer.

- l.406: this step -> these steps

Reply. Done

Reviewer #2 (Remarks to the Author):

Title and Introduction:

Based on the title, the objective of this manuscript is to identify environmental factors and human-induced pressures to study the spatial distribution of a cutaneous neglected tropical disease. Since the focus here is Buruli ulcer, it would be important to mention it in the title, as skin NTDs encompass more than a dozen diseases, all very different from one another.

It is problematic to present and discuss skin NTDs by citing Buruli ulcer, leprosy, yaws, and cutaneous leishmaniasis in the first lines of the introduction, since these diseases are caused by completely different microorganisms, with environmental or non-environmental origins, transmissible or not between humans, and requiring or not requiring a vector in their life cycle.

We understand later that the manuscript focuses on M. ulcerans, an environmentally derived pathogen. Among the other skin NTDs, some also have an environmental origin (mycetoma, chromoblastomycosis), but this is not mentioned in the introduction and no later either.

It is therefore difficult to understand the originality of this study relative to the current state of knowledge. Indeed, reference number 13 cited at the end of the introduction is titled "Mapping suitability for Buruli ulcer at fine spatial scales across Africa: A modelling study." How do the authors position themselves in relation to this study? The maps produced in the manuscript strongly resemble those presented in this 2021 publication.

Reply. Thank you for your comments, which have substantially improved the manuscript.

Methods:
Data source:

For a study of this kind, relying on diverse data involving the presence of a pathogen in the environment and/or the detection of diagnosed human cases, one would expect this section to be detailed. Instead, it refers to two supplemental tables, which themselves refer to multiple references that must then be retrieved. To understand the origin and selection of the data, one essentially must reread six additional articles. The main source of the data comes from a recent article by Simpson et al. (2019), "Mapping the global distribution of Buruli ulcer: a systematic review with evidence consensus."

Pathogeographical approach: We understand that this is the core of the analysis and the manuscript. However, for a reader who is not a modelling specialist, it is difficult to link this methods section with the two tables presented in the results and the variables listed in them.

Reply. We have modified the Data Source following your advice (lines 114-120).

We thank the reviewer for this comment. We agree that, for non-modelling specialists, the link between the Methods section and the tables presented in the Results may not have been sufficiently clear. To improve clarity, we have added a sentence in the Methods section (line 158-159) explicitly referring to the tables: "The final set of variables retained in RMUM and RBUM are presented in Tables 1 and 2". These tables summarize the variables selected by the models and complement the cartographic outputs presented in the Results section.

Results section:

The first sentences of the first and second paragraphs state:

“The RMUM distribution suggests that the most favourable areas are located in West and Central Africa (Figure 1).”

“Favourable areas for BU cases extend across West Africa, Central Africa, East Africa, and some small regions in both Northern and Southern Africa (Figure 2).”

A modelling effort is not needed to know where Buruli ulcer occurs and where *M. ulcerans* is detected. Furthermore, this is not the first modelling study to produce maps of this precision. It is difficult to understand how the authors position their work relative to the existing literature.

The results then present associations with different variables, which are discussed later, such as the presence of *L. hexandra*, a plant species, or *T. swinderianus*, a rodent. It would have been important to present information on their geographic ranges and their dynamics within the studied regions.

Reply. We agree that the geographic distribution of Buruli ulcer and *M. ulcerans* is well known, and that some previous modeling studies have produced maps. Therefore, in the revised manuscript, we have clarified how our work positions itself in relation to the existing literature.

Specifically, we now emphasize that the main contribution of our study is methodological and descriptive: (1) we explicitly integrate the environmental favourability of *M. ulcerans* into the disease model, (2) variables were preselected within categories and then combined into a single model, and (3) we incorporate new variables and their interactions that have not been considered in previous studies (e.g., Simpson et al. 2021). The originality of our work lies in expanding the scope of the study by focusing on important parameters that have never been considered before in studies, using an original modeling approach developed by our group.

Following reviewer advice, we have expanded the discussion section to provide greater ecological and geographical context for the key biotic variables identified by the models, such as *Leersia hexandra* (lines 313-316). Regarding *Thryonomys swinderianus*, information had already been provided on its known geographical distribution areas and ecological patterns within the study region (lines 401-405). These additions are intended to better contextualize our findings and clarify the added value of our modeling approach.

Discussion:
Again, this statement is problematic due to the variability among skin NTDs:

“In conclusion, our findings represent a significant advance in understanding skin NTDs by offering an approach integrating physical, bioclimatic, environmental, ecological, socio-demographic and geographical data to better understand the distribution of M. ulcerans and Buruli ulcer.”

The limitations of the modelling are appropriately noted, particularly the uncertainty, the quality of available data, and the interpretation of observed patterns. In the context of Buruli ulcer control, some African countries are more advanced than others—for example in active case detection with systematic qPCR confirmation, or in environmental detection of M.

ulcerans. Would it not be possible to validate the model results at a finer scale by selecting study areas where more reliable data are available?

Finally, the manuscript does not address the dynamics of Buruli ulcer detection rates in Africa, which are declining, whereas detection rates in Australia continue to rise. How could the authors' results explain such contrasting trends?

Reply. We here thank the reviewer concerning the fact that we appropriated discussed the limitations of our modeling approach. The reviewer is also asking two important questions. Concerning the possibility of qPCR sampling biases, with some nations being more advanced than some others, we believe that the reviewer makes an excellent point here. First, by considering only countries offering significant quality and expertise in the molecular characterization of MU and in the diagnosis of BU, we would obviously limit the number of points retained, which would therefore have an impact on the associated estimates and probabilities. Another way of doing this would be to introduce a weighting for each country into the analyses, with this weighting representing an estimate of detection capabilities—but this could also be done for research time spent and the number of researchers involved—and in this situation, certain countries, particularly Australia, would find themselves overrepresented in terms of research, according to the mean which would constitute a sampling bias. When we search, having more interest, more people, more time, and more funding to do the work, the probability of detection goes up! This is a completely different study from the work presented here, and it concerns sampling errors in research in general. Concerning the dynamics of detected rates that strongly differ between Africa and Australia, we also consider this to be another study, which does not have much to do with the work we are presenting, but which nevertheless relates to the point we discussed earlier regarding sampling bias and research effort.

Reviewer #3 (Remarks to the Author):

What are the major claims of the paper?

The authors claim that "this is the first effort to use high-resolution, continental scale spatial analyses for understanding natural and anthropogenic drivers of MU and BU distribution".

This claim is not supported as the analysis is at a coarse resolution, does not include spatial effects, does not truly identify "drivers" of either outcome, and does not adequately acknowledge earlier efforts to predict suitability for them.

Fine scale, continental level environmental suitability models for M. ulcerans and Buruli ulcer were published by Simpson et al in 2021[1]. This work is briefly mentioned in the introduction section of this manuscript but not in the discussion, which seems like a missed opportunity to compare and contrast findings.

This study also appears to rely heavily on the datasets generated through the earlier study, although the datasets themselves are not cited.

This study may improve upon the earlier study in some ways: mostly by using a more ecologically grounded approach to variable selection. However, many aspects of this work are described as though they are original, which overlooks the contribution of the earlier study.

Reply. Thank you for your remarks and thorough review, which has substantially improved the manuscript.

Examples:

1) “Limited work has been done on the regional and continental distribution of MU and BU, and when such studies do exist, they mostly estimate environmental suitability without identifying its main drivers.”

In fact, the Simpson et al study did quantify associations of environmental covariates with suitability for BU and MU using a comparable (though not identical) approach, though these associations were not interpreted to “drive” suitability. The current study does interpret correlations in this way, but without the necessary support to infer causality (or "driving"). It’s not clear how the authors are justifying their claims that environmental correlates of favourability represent drivers rather than indicators of suitability.

Reply. We thank the reviewer for this detailed and constructive comment. We agree that the comparison with Simpson et al. (2021) was not sufficiently developed in the original version of the manuscript. We have therefore added a new paragraph in the Discussion (lines 323–326) to more explicitly position our study relative to this earlier work, highlighting both the similarities in the predicted spatial patterns of favourability and the key methodological differences.

We also acknowledge that our study builds in part on datasets compiled by Simpson et al. (2021) and additional sources. While these were previously cited in the Supplementary Material, we have now cited them in the Methods section under “Data sources” to improve transparency.

With respect to the use of the term “drivers,” we agree that the associations identified by our models should not be interpreted as evidence of causality. Following the reviewer’s comment, we have revised the manuscript to consistently use the term “predictors” instead of “drivers,” unless supported by independent ecological or experimental evidence. However, the international scientific literature that is produced in disease ecology, generally use the term drivers (such as in the expression drivers of emerging infectious diseases), these drivers being associated to disease (cases) in a linear way or in a more complex non-linear one.

We have further clarified in the Discussion the contribution, improvement, and distinction of our work relative to previous studies (lines 283-288). Specifically, we emphasize: (1) the inclusion of novel variables and interaction terms that have not been jointly considered in previous studies, (2) the pre-selection of variables within thematic parameter categories prior to their integration into a single model, and (3) the explicit integration of *M. ulcerans* environmental favourability into the Buruli ulcer disease model. According to our known

knowledge of the scientific literature this is the first time, at least for this type of infection, that a conditionality (i.e., the presence of the bacterium in the environment) is introduced to model disease cases.

These revisions aim to better acknowledge prior work, clarify the added value of our approach, and distinguish between statistical associations and potential causal interpretations.

2) "To our knowledge these are the first models demonstrating MU spatial distribution consistently correlated with environmental macro-environment conditions"

It's not clear what the authors are aiming to say here. I interpret this to mean that correlations between favourability score and macro-environmental covariates are consistent across space, but there is no evidence from results shown in the manuscript to support this. In fact the scatterplots in Fig S1. seem to contradict this: correlations between favourability scores and environmental covariates vary between levels of other covariates.

The Simpson et al study also showed associations of MU with macro-environmental covariates, and the overlap in the factors identified would be interesting to elaborate on in the discussion.

The authors further state: "Using a relatively fine spatial scale of resolution, 88×88 km2 and an extensive dataset of independent parameters, we show how natural and anthropogenic factors influence the current geographic ranges of MU and BU cases."

The resolution of the Simpson et al study was 5x5km; i.e. almost 300x higher than in this study, so the claim that this is "relatively fine" is not supported. A resolution of 88km2 is unsuitable for modelling the distribution of such a focal disease. Its extreme localisation within endemic countries suggests that ecological drivers of MU & BU operate on a much finer scale via local interactions and effects. Evidence from several sources is summarised in the introduction of Landier et al [2].

Presumably due mostly to the coarse resolution used, predicted favourability for BU is very broad (covering most of the areas represented by occurrence records) and I question the public health utility of the finding that all regions of 6 countries are highly favourable for the disease. Given that BU is highly focal, with low and declining incidence, how will these findings support its control?

I believe that the coarse resolution of analysis is the main flaw in this study.

Reply. We thank the reviewer for this detailed comment. We would like to clarify that our choice of grid resolution was motivated by several factors. First, it is currently not feasible to compile the full set of environmental and anthropogenic variables we included at finer resolutions across continental Africa. Second, certain macroecological and anthropogenic processes influencing *M. ulcerans* and BU occurrence operate at broader spatial scales and may not be captured in very fine-scale analyses. In other words, not considering effects that occur on a larger scale than the individual, i.e. susceptible people, or the village, e.g., the communities of people and their habits and behaviours, means neglecting these possible and

entirely conceivable influences, e.g., the role of an invasive plant in surrounding areas. This is what we refer to in our research field as scaling issues.

We also note that using a finer grid with relatively few presence points would increase the number of zeros in the response, potentially leading to overfitting and unstable model predictions. Therefore, our approach represents a compromise, also called a trade-off, of very fine scale but loosing parameter information and diversity or large scale but loosing local specificities also called idiosyncrasies, that allows continental-scale coverage while maintaining robust statistical inference. In addition, the reviewer is refereeing to Landier et al.' work writing that ecological drivers of MU & BU operate on a much finer scale via local interactions and effects. We do not know if the reviewer is aware of this, but as one of the authors is one of our co-authors (J-FG), Landier et al.' work focused on understanding what was happening in Akonolinga, Cameroon, in an area about 60 × 60 km, because he had no other data available for his *PhD* thesis. The student was originally supposed to work on a national scale, which was not possible. A nationwide study would have revealed other variables and predictors to explain MU and BU, as the study and its results were heavily influenced by restricted access to data.

While the study of Simpson et al. (2021) used a finer resolution (5×5 km), this does not imply that all subsequent studies must be conducted at the same scale. Importantly, the spatial patterns predicted by our models are broadly similar to the spatial patterns predicted described by Simpson et al., supporting the validity of our approach. In addition, scaling-up in our study permits us to consider other potential variables and predictors (and even drivers) that were not considered by previous finer-scale studies like in Simpson et al.

Our study further contributes by including a broader set of environmental and anthropogenic variables, as well as their interactions, which have not been jointly considered in previous studies. Even at a coarser resolution, these variables provide insight into the macro-scale predictors of *M. ulcerans* and BU occurrence that complement finer-scale analyses in understanding disease ecology.

Abstract
The abstract is not scientific; it overstates the significance of the work and does not describe the methods or the key results.

Reply. We completely agree, and we now have modified the abstract in the submitted version.

Discussion
False claims: "Although disease caused by MU has never been described in wild or domestic animals in Africa, this may reflect a lack of attention to the presence of the disease in animal hosts in general" MU has been identified in clinical samples from lesions of several domestic animals in Benin 3

Vague conclusions: “Expanding this type of research to other cutaneous infectious and parasitic diseases will help advance global public health by accounting for global environmental changes” What type of research are you advocating for? Which infections/ diseases? How can this account for global environmental changes?

Reply. We completely agree with the reviewer that MU lesions have been found in domestic animals, notably in Africa, and we have now modified the text accordingly and citing the reference as proposed by the reviewer. We thank the reviewer for this important comment.

To be added in our text: Djouaka, Rousseau, et al. "Domestic animals infected with Mycobacterium ulcerans—Implications for transmission to humans." PLoS neglected tropical diseases 12.7 (2018): e0006572.

We also agree with the reviewer that we stayed vague in our previous discussion concerning the type of we are advocating for, the categories of infection well suited for, and the impacts exerted by global changes on those types of diseases. We now better explain these ideas in the new document sent for revision to the journal.

Further issues

Supplementary:
More information is needed about “R2-partial” values; I assume these should be described as partial R2

Figure legends should be wholly self-explanatory, and should not state “See main text for further explanation”. What is HN-Surface?

Reply. We have revised the main text and the Supplementary Information to use the standard notation “partial R^2” consistently.

We have added to the legend that it is Humidity near Surface, i.e., HN-Surface, which is the mean near-surface relative humidity (%). It is also added to the main text the first time it is mentioned, as well as in the table of variables in the supplementary material.

Appropriateness and validity of any statistical analysis

There is no information about how absences were identified. If simply absence of occurrence, they should consider how under detection may lead impact findings.

The authors appear to have indiscriminately allowed for interactions between selected variables (p13 lines 363- 365): “After this selection process another model was fitted, incorporating both the selected variables and their two-way interactions, resulting in the refined Buruli ulcer Model (RBUM)”. This tolerance for interaction terms has led to dominance of drivers which are very difficult to interpret: e.g. “approximately 29.3% of the variation in the BU model was explained by sunlight radiation seasonality (UVB 2) interacting with climatic and edaphic conditions… Additional but less influential factors (accounting for 6% of BU cases 284 overall) included the interaction between BIO-6 and the rodent *Thryonomys swinderianus*.”

It is generally recommended that interaction terms should be allowed only when there is prior evidence or theoretical support for a non-additive relationship.

Reply. The formalism of interaction use is broader than that discussed by the reviewer. There is also a difference in the use of these interactions in experimental laboratory work and statistical analyses of the same type as those we have produced. The *a priori* introduction of interactions and their factors (simple variables are then called factors) serves to show whether it is the simple variables or some of them that are explanatory, or whether it is interactions between factors; in such a situation this tends to indicate that the explanation of the dependent variable is complex, better explained by variable products and also threshold effects, as we show in this paper. Normally, for many infectious agents and their transmission, here MU and BU respectively, the introduction of interaction terms should therefore be recommended, which is rarely done due to a preconceived notion of seeking simple explanations based on single variables.

Reproducibility
Possible, although neither study datasets nor code are provided.

Reply. We have included a table with the presences of *M. ulcerans* and Buruli ulcer cases in the study area.

1 Simpson, Hope, et al. "Mapping suitability for Buruli ulcer at fine spatial scales across Africa: a modelling study." PLoS neglected tropical diseases 15.3 (2021): e0009157.

2 Landier, Jordi, et al. "Spatio-temporal patterns and landscape-associated risk of Buruli ulcer in Akonolinga, Cameroon." PLoS neglected tropical diseases 8.9 (2014): e3123.

3 Djouaka, Rousseau, et al. "Domestic animals infected with Mycobacterium ulcerans—Implications for transmission to humans." PLoS neglected tropical diseases 12.7 (2018): e0006572.

Reply. We thank the reviewer for citing these three papers, noting that we cited Simpson et al.'s work in our previous version. We now cite also Djouka et al.' paper.

REPLIES TO REVIEWERS

Referee expertise:

Referee #1: Disease Ecology, Climate, Spatial Modeling

Referee #2: Buruli Ulcer, Epidemiology

Referee #3: Buruli Ulcer, Skin NTDs, Africa, Epidemiology

Reviewers' comments:

Reviewer #1 (Remarks to the Author):

I have now reviewed the revised manuscript by Dr. Aliaga-Samanez and colleagues. I believe the authors have done a good job of addressing my comments and updating the manuscript accordingly. In particular, I appreciated the clarifications regarding the model's validation and the interpretability of the results.

However, I am less convinced by the authors' response regarding the extrapolation of the BU model to pantropical regions outside those used for training in Africa. Perhaps my comments were not entirely clear in the previous review stage (apologies if that was the case), but I feel the authors have conflated two distinct points in their response.

The first point referred to the reportedly exponential relationship between temperature and pathogen favorability (line 215 in the revised manuscript). While I agree with the authors that, in this context, an exponential function may not be the most biologically interpretable (plus, the forced capping at one may not be the most elegant solution in purely mathematical terms), if it fits the data, that is fine with me.

The second point referred to the section titled 'Extrapolating the African BU model to tropical and subtropical regions worldwide' (line 266 in the revised manuscript). It is clear from this section that the authors are using their RBUM, which is an explanatory model for reported cases. I would not have made this point if the authors had used their RMUM for extrapolation; once uncertainty is accounted for, this is a standard exercise in habitat distribution modelling. The problem with the RBUM is that case distribution depends on several context-dependent socioeconomic factors (the exposure and vulnerability components of the risk equation that I mentioned) that cannot simply be treated in the same way as environmental or climatic variables. This is why I suggested interpreting the results shown in Figure 4 with some caution.

Reply. We thank the reviewer for his/her feedback. First, the RBUM model is conditioned by the fact that we impose the results of the RMUM model as an explanatory condition, that is, what explains the distribution of the mycobacterium *M. ulcerans*. Put another way, the development of cases is imposed by the presence of the bacillus in the same space, which already limits the possibility of producing risky extrapolations.
Second, we are fully aware that individual and community exposures and vulnerability are very important to consider. And that is precisely why we are

writing in the discussion that our intertropical extrapolation of BU needs to be taken with caution. However, the presence of certain variables, such as the development of invasive plants (*L. hexandra* and *E. pyramidalis*), and rainfed croplands corresponds to environments that are generally human-modified, and occupied by humans for their agricultural activities, for example. We are nevertheless aware that an analysis of the risk of infection with *M. ulcerans* should consider exposures and vulnerabilities, which could be a possible area of study for other authors based on the work we are proposing.

Reviewer #2 (Remarks to the Author):

All my previous comments regarding the introduction have not been adequately addressed, despite the statement in the point-by-point responses: "Thank you for your comments, which have substantially improved the manuscript." The main issue remains that the current version does not clearly present the state of the art or the specific added value of this manuscript compared with previous studies.

Reply. Thank you for your comment. We had already included more detailed information about the state of the art of our paper at the beginning of the Discussion, but we have now also added this to the Introduction and removed the sentence you mentioned below.

Furthermore, the sentences at the end of the introduction — "We discuss how to leverage interoperable databases to better understand disease patterns and processes acting at different spatial and temporal scales. By doing so, other skin NTDs, and more widely NTDs, can be studied in a similar way. Ultimately, this approach can enhance prevention and control strategies by identifying key environmental risk zones and vulnerable populations" — do not seem appropriate for this section. These statements are highly speculative and would be more suitable for the discussion.

Reply. We agree with the reviewer. These statements have now been removed from the Introduction, and are now placed in the discussion section.

Regarding the comment raised during the review process:

"Finally, the manuscript does not address the dynamics of Buruli ulcer detection rates in Africa, which are declining, whereas detection rates in Australia continue to rise. How could the authors' results explain such contrasting trends?"

The response provided is not satisfactory. It is difficult to claim that a model

identifies factors likely to explain the presence of MU/BU without incorporating temporality and explicitly addressing the epidemiological dynamics of the disease. Ignoring these temporal trends considerably limits the explanatory power and relevance of the proposed model.

Reply. We thank the reviewer for raising this important point. We agree that contrasting temporal trends in Buruli ulcer detection between regions such as Africa and Australia are epidemiologically relevant.

However, this aspect falls beyond the scope of the present study. Our study was designed as a cross-sectional spatial modelling analysis aimed at identifying environmental suitability and associated factors for *M. ulcerans* and Buruli ulcer occurrence, rather than explicitly modelling temporal trends in detection rates, and discuss the reasons why Victoria, Australia is seeing an increase of BU cases and many African countries the converse situation. The explanations could be numerous, particularly for some African countries, e.g., a lack of surveillance, e.g., several African countries, but also a lack of diagnostic skills, e.g., difference between French Guiana and surrounding countries. This is a completely different matter, outside the scope of our own work.
We note in the Discussion that future studies could benefit from incorporating temporal data to better understand contrasting epidemiological trends across regions, but also from a constant and sustained monitoring and research effort, because that is also a possible explanation to “why more, and why less”.

Reviewer #3 (Remarks to the Author):

The authors have mostly addressed the key points raised though there are two outstanding issues.
1. I agree with the Reviewer 2 comment that Buruli ulcer should be included in the title but this change has not been made.

Reply. We thank the reviewer. We have modified the title following the suggestion.

2. The authors have soundly explained their reasoning for the use of an 88km2 grid. However the first line of the discussion still states “To our knowledge, this is the first effort to use high-resolution, continental scale spatial 278 analyses for understanding natural and anthropogenic drivers of the spatial 279 distribution of Mycobacterium ulcerans and Buruli ulcer cases.” I do not think that any experienced reader would consider the scale of the models “high-resolution”. I suggest they drop this descriptor.

Reply. Thank you for this comment. We have now removed "High resolution".
