# Supplementary material for: Human-induced pressures and environmental factors drive the distribution of neglected tropical disease Buruli ulcer
Source: Commun Med (Lond). 2026 Jun 4;6:536. doi: 10.1038/s43856-026-01692-6 (PMC13672292; doi:10.1038/s43856-026-01692-6)
Supplement: Supplementary file 2 [file 43856_2026_1692_MOESM2_ESM.pdf]

Supplementary Information

Human-induced pressures and environmental factors drive the distribution of neglected tropical disease Buruli ulcer

Alisa Aliaga-Samanez[1,2], Marina Cobos-Mayo[3], Christine Chevillon[1], Magdalene Dogbe[4], Kayla M. Fast[5], Matthew E. Scott[5], Kelly Waters[6], Joseph P. Receveur[7], Jennifer L. Pechal[6], Michael W. Sandel[5,8], Heather R. Jordan[4], M. Eric Benbow[6,9,10,11] and Jean-François Guégan[1]

1. MIVEGEC, UMR Université de Montpellier, CNRS, IRD, Montpellier, France.
2. Instituto de Investigación en Recursos Cinegéticos (IREC – CSIC, UCLM), Ciudad Real, Spain
3. Grupo de Biogeografía, Diversidad y Conservación, Departamento de Biología Animal, Facultad de Ciencias, Universidad de Málaga, Málaga, España.
4. Department of Biological Sciences, Mississippi State University, MS, USA.
5. Department of Wildlife, Fisheries and Aquaculture, Mississippi State University, MS, USA.
6. Department of Entomology, Michigan State University, East Lansing, MI, USA.
7. Institute for Genome Sciences, University of Maryland Baltimore, Baltimore, MD, USA.
8. Forest and Wildlife Research Center, Mississippi State University, MS, USA.
9. Department of Osteopathic 22 Medical Specialties, Michigan State University, East Lansing, MI, USA.
10. Ecology, Evolution and Behavior Program, Michigan State University, East Lansing, MI, USA.
11. AgBioResearch, Michigan State University, East Lansing, MI, USA.

Table of Contents

Table S1. Favourable regions for *Mycobacterium ulcerans* by country. 3

Fig. S1. Relationships between favourability for *M. ulcerans* (MU Fav) and significant independent parameters as presented in the corresponding statistical model 4

Table S2. Favourable regions for Buruli ulcer by country. 6

Fig. S2. Relationships between favourability for Buruli ulcer cases (BU Fav) and significant independent parameters as presented in the corresponding statistical model 7

Table S3. Dependent variables used for distribution modelling 10

Table S4. Independent variables used for distribution modelling. 11

Model fit assessment for Africa 14

Table S5 Model fit assessment based on discrimination and classification capacities respect to MU and BU records. 14

Table S6 Validation of model predictive capacity based on discrimination and classification performance respect to disease records 14

Supplementary References 15

Table S1. Favourable regions for *Mycobacterium ulcerans* by country.

This table summarizes the environmental favourability for *M. ulcerans* in African countries based on a spatial distribution model. The classes (e.g., low, intermediate, high) reflect the favourability of environmental conditions for the presence of the pathogen. When available, specific regions within countries (e.g., north, south) are indicated to show where these favourability levels occur. When a given country is not listed means that favourability is null.

Country	Favourability for MU	Region
**Nigeria**	Intermediate to High	Southern region
**Côte d'Ivoire**	Intermediate to High	Southern region
**Benin**	High (south), Intermediate (north)	Southern region favorable, northern area mentioned separately
**Ghana**	High (south), Intermediate (north)	Southern region favorable, northern area mentioned separately
**Togo**	Intermediate to High	Southern region
**Democratic Republic of Congo**	Low to Intermediate/High	Concentrated in the central region of the Zaïre-Oubangui drainage area
**Somalia**	Intermediate to High	Coastal regions
**Kenya**	Intermediate to High	Coastal regions and near Lake Victoria
**Tanzania**	Intermediate to High	Coastal regions high, central and southern intermediate
**Uganda**	Intermediate to High	Various areas, especially near lakes
**Ethiopia**	Low to Intermediate	Patchy favourability areas
**Eritrea**	Intermediate to High	Patchy favourability areas
**Burkina Faso**	Intermediate	Southern region (Patchy favourability areas)
**Mali**	Intermediate	Southern region (Patchy favourability areas)
**Guinea**	High	Northeast area (Patchy favourability areas)
**Gambia**	Intermediate	Southeast region
**Senegal**	High	Southern region (Patchy favourability areas)
**Malawi**	Intermediate to High	Northern regions
**Angola**	High	Only in Western region

Fig. S1. Relationships between favourability for *M. ulcerans* (MU Fav) and significant independent parameters as presented in the corresponding statistical model (see Table 1 in the main text). Only significant variables with a threshold value of partial R^2 ≥0.03 (i.e., 3%) are illustrated. Panel **a** shows the relationship between MU Fav and the two-way interaction term between the minimal temperature of the coldest month (BIO-6) and the humidity near soil surface (HN-Surface) categorized in three classes (Partial R^2=0.356, p<0.0001). Panel **b**, the relationship between MU Fav and the two-way interaction term between BIO-6 and the absence or presence of *Leersia hexandra (*Partial R^2=0.123, p<0.0001). Panel **c**, the relationship between MU Fav and the two-way interaction term between the diversity in number of soil types and the absence or presence of gold in soils *(*Partial R^2=0.038, p<0.0001). See main text for further explanation. Details are given here of the explanatory variables in the MU model (see main text, Table 1). In our MU statistical investigation, we identified the presence of gold in soils and subsoils as favourable conditions for MU. Gold mining (and arsenic; see Duker and colleagues[1]) is suspected to be a risk factor practice for contracting MU and other infectious diseases[2], and Keita and colleagues[3] revealed a significant spatial correlation between BU foci and gold-panning sites in Guinea, western Africa. This correlative trend should be a supply-side effect of a lack of occupational safety and hygiene measures (e.g., sharp instruments causing cutaneous lesions, silicate minerals present in gold veins responsible for skin lesions) for gold miners. The use of water lances, in particular, to wash the soil and release the gold particles would at the same time facilitate the dispersion of mycobacteria whose origin is suspected to be the rhizosphere of certain plants or certain types of soil[4]. The development of MU in aquatic and subaquatic environments could thus be directly due to the extraction of gold and the release of mycobacteria associated with degraded soil or the exposed rhizosphere of plants.

In addition, several bacterial taxa can actively contribute to the formation of gold grains in surface environments[5], and MU and other MTNs taxa could benefit from such conditions being actively or non-actively involved in the biogeochemical cycling of such rare and precious metals. Some minerals, including gold, widely found in clay soils can facilitate the growth of mycobacteria[6], with bacteria interacting and affecting clay minerals though aggregation, flocculation and also dissolution, sometimes accelerating mineral transformation[7]. Iron, lead and magnetite are heavier metals and minerals that are often associated with the presence of gold in subsoils, and so the extraction of gold would be part of the disorganization of an entire mineral and organic ecosystem. The possibility of interaction between this type of non-tuberculous mycobacteria and gold particles remains an open question.

In tropical regions, warm and humid climates tend to intensify plant growth and organic matter decomposition, thus producing organic acids which cause soil acidification[8]. In boreal regions, the presence of coniferous trees and mosses can contribute to the development of acidic conditions in soils and freshwaters[8]. Soil *pH* is influenced by both local and regional factors, including vegetation type, soil development stage and local temperature and precipitation, and these parameters contribute to the diverse *pH* conditions observed in soils across various climatic environments. As a result, different bacterial taxa may exhibit different *pH* preferences[8]. In tropical, subtropical, boreal, and temperate forests as well as in arctic

tundra, bacterial genera with low *pH* optima predominate due to the acidic nature of their soils. Concerning MU, which belongs to Actinomycetota (or Actinobacteria), both field and experimental studies identified *pH* as one of the key parameters delineating the distribution of this bacterium in tropical regions, with a *pH* range observed between 5.30 and 6.30, even if more acidic conditions may be possible[9–11]. In the present study, we failed to show an effect of *pH* on the spatial distribution of MU in Africa. *pH* is admitted as a primary and consistent factor structuring soil bacterial diversity and community composition[12].

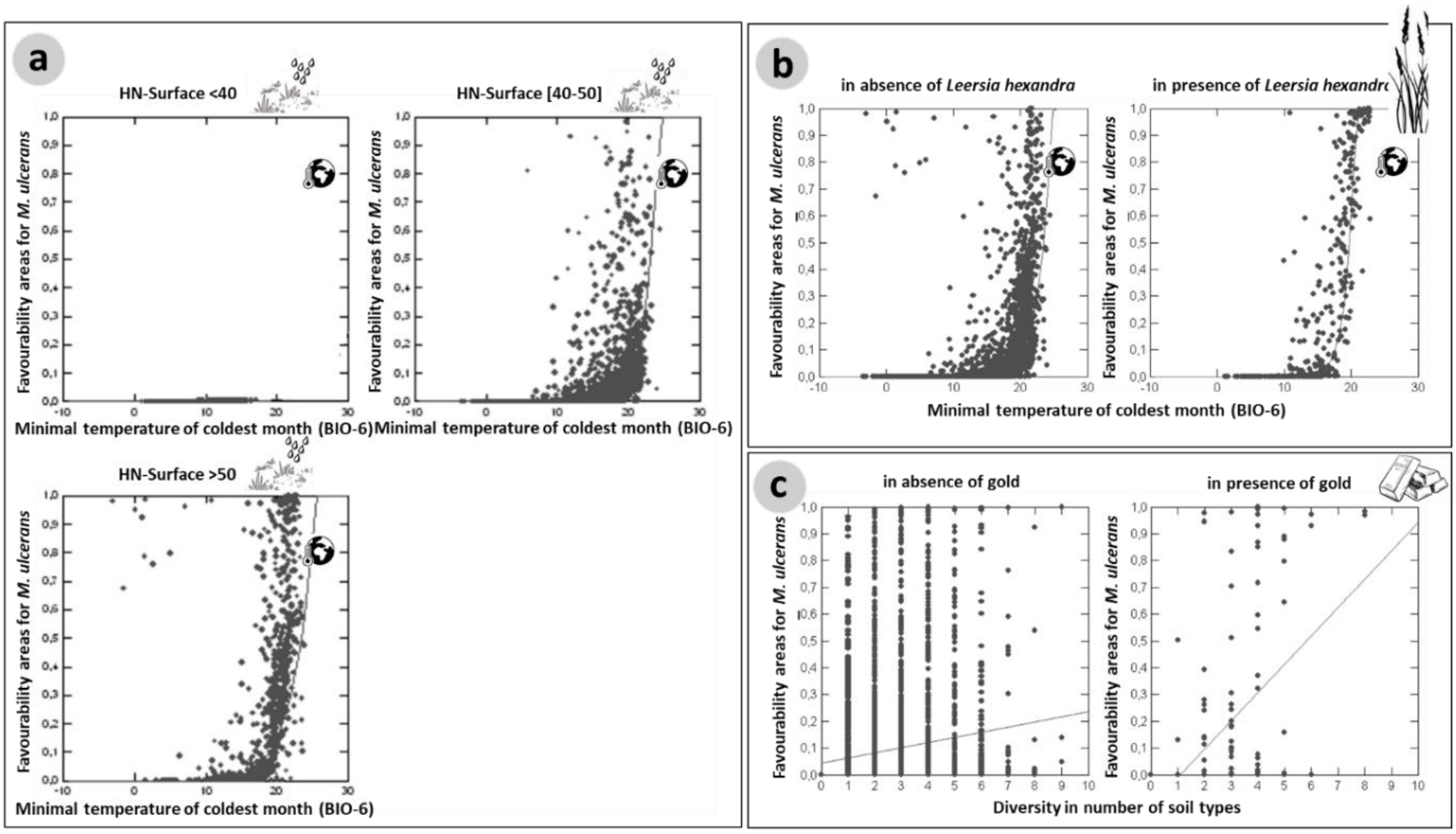

Table S2. Favourable regions for Buruli ulcer by country.

This table summarizes the environmental favourability for Buruli ulcer in African countries based on a spatial distribution model. The classes (e.g., low, intermediate, high) reflect the favourability of environmental conditions for the presence of the pathogen and reported cases. When available, specific regions within countries (e.g., north, south) are indicated to show where these favourability levels occur. When a given country is not listed means that favourability is null.

Country	Favourability for BU	Region
**Sierra Leone**	High	All regions
**Liberia**	High	All regions
**Côte d'Ivoire**	High	All regions
**Ghana**	Intermediate to High	All regions
**Benin**	High	All regions
**Togo**	High	West Africa
**Nigeria**	Intermediate to High	Southern region
**Cameroon**	Intermediate to High	All regions
**Equatorial Guinea**	High	All regions
**Republic of the Congo**	Intermediate to High	All regions
**Democratic Republic of Congo**	Intermediate to High	Almost the whole region
**Central African Republic**	Intermediate to High	All regions
**Uganda**	Intermediate to High	Almost the whole region
**Kenya**	Intermediate to High	Patchy favourability areas
**Tanzania**	Intermediate to High	Patchy favourability areas
**Algeria**	Low to Intermediate	Northern regions
**Morocco**	Low to Intermediate	Northern regions
**Egypt**	Low to Intermediate	Near Nile River and other areas
**Malawi**	Intermediate	Southern regions
**Zimbabwe**	Intermediate	Patchy favourability areas
**South Africa**	Intermediate	Humid subtropical region
**Niger**	Low to Intermediate	Only western region
**Senegal**	Low to Intermediate	Patchy favourability areas
**Chad**	Intermediate	Borders with Nigeria, Cameroon, Central African Republic
**Botswana**	Intermediate	Only some regions in the south
**Lesotho**	Intermediate	All regions
**Eswatini**	Intermediate	All regions

Fig. S2. Relationships between favourability for Buruli ulcer cases (BU Fav) and significant independent parameters as presented in the corresponding statistical model (see Table 2 in the main text). Only significant variables with a threshold value of Partial R^2 ≥0.03 (i.e., 3%) are illustrated. Panel **a** shows the relationship between BU Fav and the two-way interaction term between the annual ultraviolet B radiation seasonality (UVB-2) and minimal temperature of the coldest month (BIO-6) categorized in 4 classes (Partial R^2=0.293, p<0.0001). Panel **b**, the relationship between BU Fav and *Mycobacterium ulcerans* model *logit* values (Partial R^2=0.279, p<0.0001). Panel **c**, the relationship between BU Fav and the two-way interaction term between soil moisture and altitudinal elevation, with 4 classes of altitude (Partial R^2=0.186, p<0.0001). Panel **d**, the relationship between BU Fav and the two-way interaction between distance to a populated area and rainfed croplands divided in 2 classes (Partial R^2=0.072, p<0.0001). Panel **e**, the relationship between BU Fav and the two-way interaction between BIO-6 and the absence or presence of *Thryonomis swinderianus* (Partial R^2=0.068, p<0.0001). Panel **f**, the relationship between BU Fav and the presence of acrisols in the area (Partial R^2=0.061, p<0.0001). Panel **g**, the relationship between BU Fav and the absence or presence of *Echinochloa pyramidalis* (Partial R^2=0.052, p<0.0001). Panel **h**, the relationship between BU Fav and the two-way interaction between distance from a populated center and altitudinal elevation categorized in 4 classes (Partial R^2=0.032, p<0.0001). Panel **i**, the relationship between BU Fav and the two-way interaction between UVB-2 and soil type diversity in the area in 4 different classes (Partial R^2=0.031, p<0.0001). See text for further explanation.

Interestingly, a third invasive plant species, *Commelina benghalensis*, also appeared in BU analyses but was ultimately not retained in minimal BU model. As *L. hexandra* and *E. pyramidalis*, this third invasive plant is also a metal-hyperaccumulator, with such shared property having recently reinforced the human interest for the use of all three plants in new green industrial processes[13–16]. In addition, a study performed across nearby rural, peri-urban and urban wetlands in Cameroon brought results supporting, in one hand, a high similarity in soil preferences among these three invasive plants, and, in the other hand, an even enhanced abundance of *C. bengalensis* relatively to both invasive grass species in peri-urban areas[17]. Therefore, the absence of *C. bengalensis* in our statistical models might relate to the redundancy of information brought by this species and the information already present and/or the socio-economic factor pointing the peri-urban areas. Altogether, the effects of the three plant species on MU and BU spatial distribution raise the point of the human impacts that directly (through human-use for livestock or phytoremediation) or indirectly (through pollution favouring those plants) maintain landscapes favoring MU persistence near human settings and MU contacts with human derma. See main text.

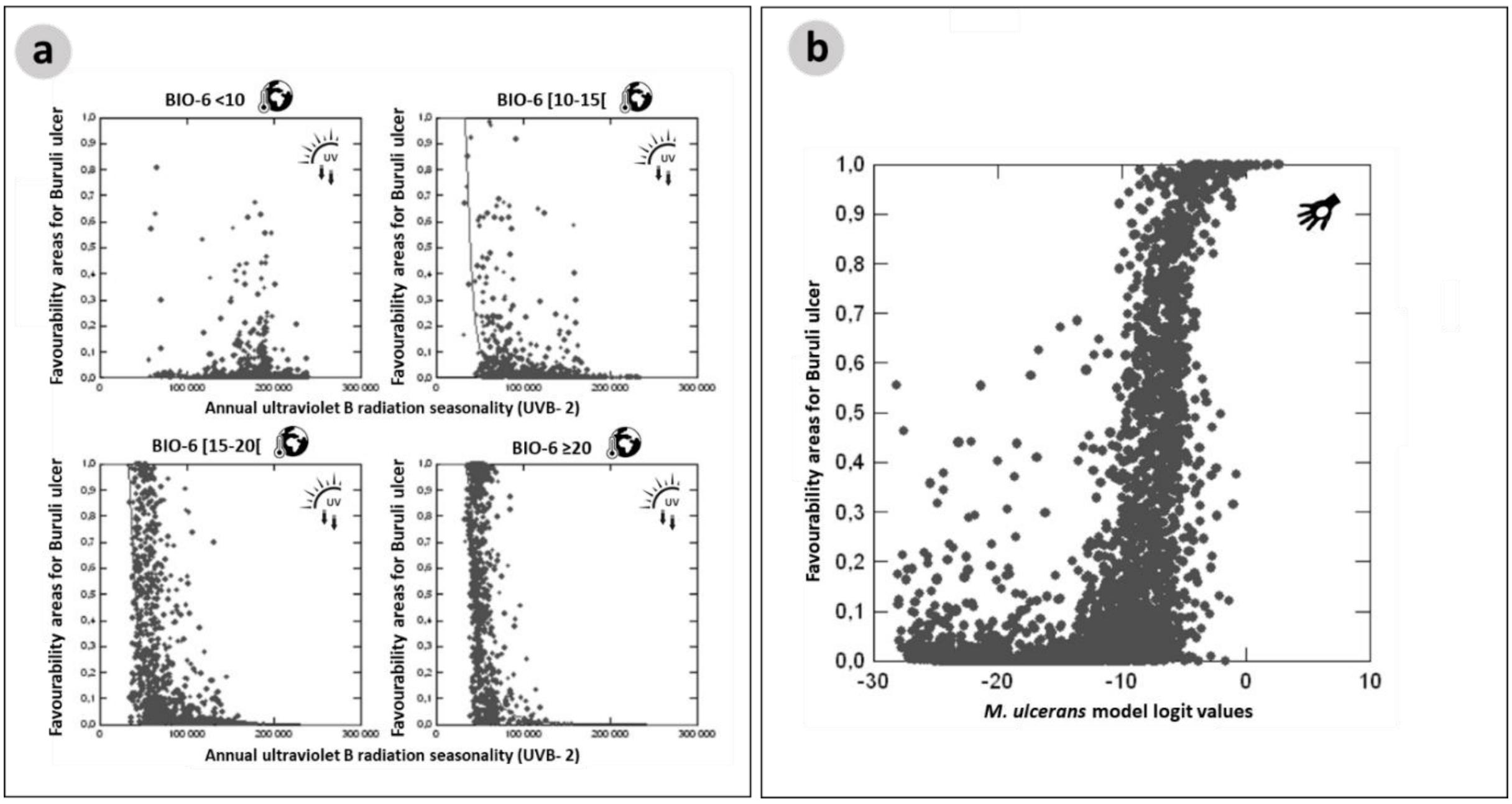

a
BIO-6 <10
BIO-6 [10-15[
BIO-6 [15-20[
BIO-6 ≥20
Favourability areas for Buruli ulcer
Annual ultraviolet B radiation seasonality (UVB- 2)
UV
b
Favourability areas for Buruli ulcer
M. ulcerans model logit values

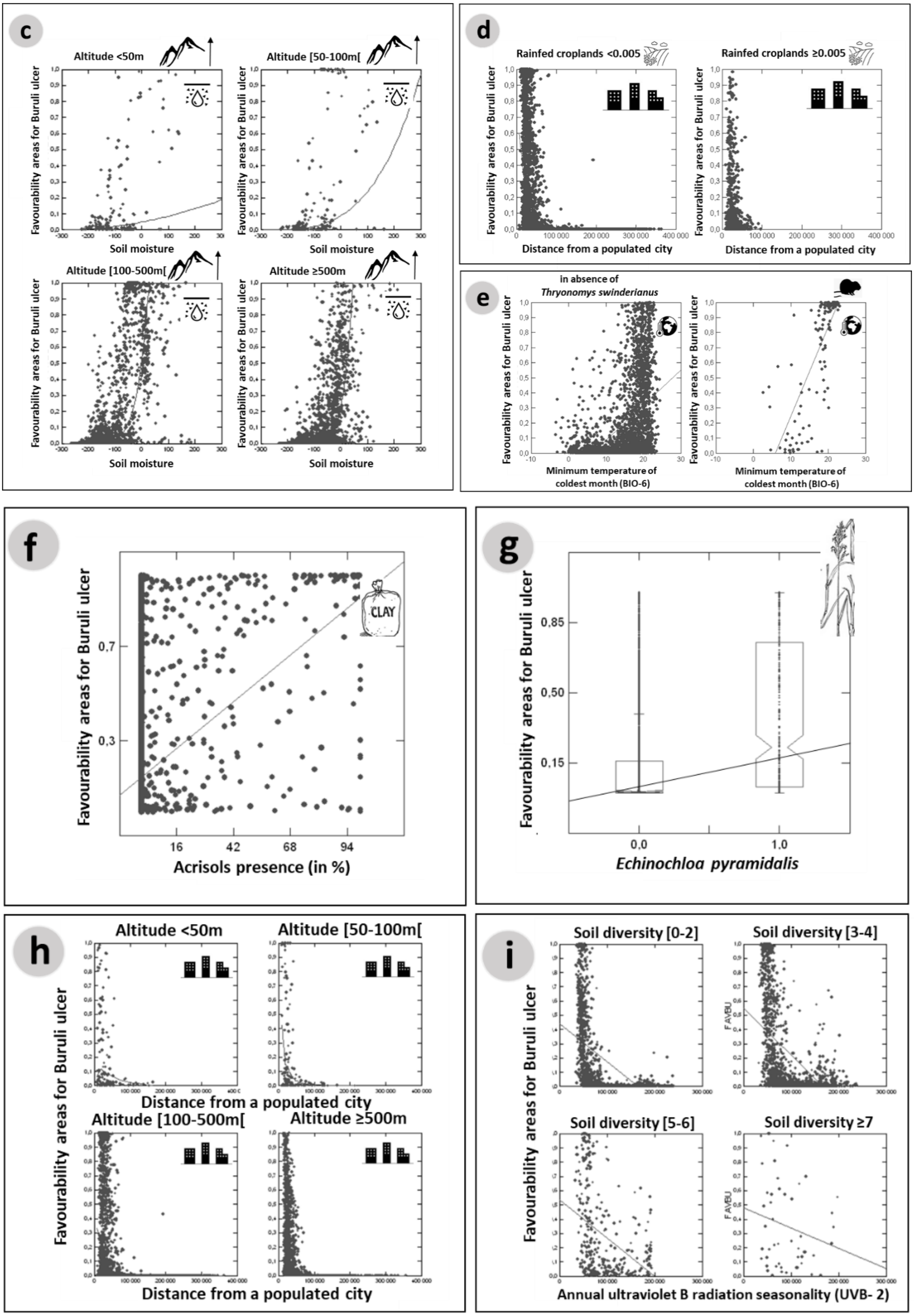
c
Altitude <50m
Altitude [50-100m[
Altitude [100-500m[
Altitude ≥500m
Favourability areas for Buruli ulcer
Soil moisture
d
Rainfed croplands <0.005
Rainfed croplands ≥0.005
Favourability areas for Buruli ulcer
Distance from a populated city
e
in absence of
Thryonomys swinderianus
Favourability areas for Buruli ulcer
Minimum temperature of coldest month (BIO-6)
f
CLAY
Favourability areas for Buruli ulcer
Acrisols presence (in %)
g
Favourability areas for Buruli ulcer
Echinochloa pyramidalis
h
Altitude <50m
Altitude [50-100m[
Altitude [100-500m[
Altitude ≥500m
Favourability areas for Buruli ulcer
Distance from a populated city
i
Soil diversity [0-2]
Soil diversity [3-4]
Soil diversity [5-6]
Soil diversity ≥7
Favourability areas for Buruli ulcer
Annual ultraviolet B radiation seasonality (UVB- 2)

Table S3. Dependent variables used for distribution modelling

Sources of data on the presence of *Mycobacterium ulcerans* in water and soil and reported human cases of Buruli ulcer.

Type of Data	Sources
Mycobacterium ulcerans* (MU)	[18–20]
**Buruli ulcer (BU) human cases****	[18,20–24]

*The georeferenced data for MU occurrence in Africa cover a 36-year period (1982-2018)

**The georeferenced data for human BU cases in Africa cover a 60-year period (1957 to 2017)

Table S4. Independent variables used for distribution modelling.

A total of 110 variables, grouped into five general sets, were used for modelling. Also, the two-way interaction terms between predictor variables of any group were included in the modelling procedure. The taxa selected in the animal and the plant diversity groups respond to previous knowledge on the ecology of *Mycobacterium ulcerans*. The variable units are indicated in parenthesis. Concerning marsupials, since our MU model was trained on African data where this taxonomic animal group does not exist, we did not consider them in further extrapolated models. Interestingly, in absence of marsupials in our model, we were able to extrapolate the distribution of BU in Australia fairly accurately based on the cases observed. For insects potentially acting as vectors, we initially considered the three genera *Aedes, Anopheles and Culex*, but due to their near global distribution, we did not consider them in further analysis. Considering species rather than genera generated too many variables.

Group	Factor	Variable codes and definition	Source
PHYSICAL	Mineral presence	Metallic and non-metallic mineral resources (presence and absence):<br>- Al: aluminium - As: arsenic<br>- Au: gold - Ca: calcium<br>- Cu: copper - CO_2: Dioxide of carbon<br>- Fe: iron - GYP: gypsum-anhydrite<br>- Hg: mercury - K: potassium<br>- LST: limestone - LST_C: limestone-High<br>- Mn: manganese - N: nitrogen<br>- P: phosphates - Pb: lead<br>- S_A: sulphur - S_P: sulphur-pyrite<br>- Sb: antimony - Se: selenium<br>- U: uranium - Zn: zinc	25
		$CaCO_3$: Calcium carbonate (% of weight)	26
		GYP: Gypsum (% of weight)	
		Total_C: Total inorganic carbon (% of weight)	
		Total_K: Total potassium (% of weight)	
		Total_N: Total nitrogen (% of weight)	
		Total_P: Total phosphorus (% of weight)	
		Total_S: Total sulfur (% of weight)	
	Texture	Clay content (% of weight)	26
		Bulk density (g/cm^3)	
		Gravel content (% of weight)	
		Sand content (% of weight)	
		Silt content (% of weight)	
	Topography	Altitude (m)	27

		Slope (decimal degrees)	
	Exposure of fresh water	Dist_River: distance to fresh waters (meter)	28
**EDAPHIC AND CLIMATE**	Carbon density	BE_biomass: belowground biomass (Mg C/Ha)	29
	Soil diversity	Number of soil types by hexagon unit of 88×88km^2	30
	Diversity of dominant soil	Richness of the dominant soil types by hexagon unit of 88×88km^2:<br>-Acrisols -Histosols -Rankers -Andosols<br>-Kastanozems -Regosols -Arenosols -Lithosols<br>-Rendzinas -Cambisols -Lluvisols -Solonchaks<br>-Chernozems -Nitosols -Solonetz -Ferralsols<br>-Phaeozems -Vertisols -Fluvisols -Planosols<br>-Xerosols -Gleysols -Podzols -Yermosols<br>-Greyzems -Podzoluvisols	30
	Cation exchange capacity	*pH*	26
		ECE: Electrical conductivity (ds/m)	26
		Exchangeable cations (cmol/kg):<br>- EXCA: calcium - EXMG: magnesium<br>- EXK: potassium - EXNA: sodium<br>- EXAL: aluminium - CEC: cation exchange capacity	
	Temperature	BIO-1: Mean annual air temperature (°C)	31
		BIO-5: Mean daily maximum air temp. of the warmest month (°C)	
		BIO-6: Mean daily minimum temp. of the coldest month (°C)	
		BIO-7: Annual range of air temp. (°C)	
	Precipitation	BIO-12: Annual precipitation amount (kg m^{-2} year^{-1})	31
		BIO-15: Precipitation seasonality (kg m^{-2})	
	Humidity	Moisture: mean moisture (kg m^{-2} year^{-1})	
		HN Surface: mean near-surface relative humidity (%)	
	Ultraviolet Solar Irradiance	UVB_1: Annual mean UV-B (4,000 – 8,600 J/m^2/day)	32
		UVB_2: UV-B seasonality (standard deviation) (3,100 – 310,000 J/m^2/day)	

**ANIMAL DIVERSITY**	Diversity of native/introduced animals	Species richness and/or presence-absence by hexagon unit of 88×88km^2:<br>→ Fish genus: *Gambusia*, *Oreochromis, Osteoglossum,* and *Poecilia*<br>→ Insect genus: *Aedes, Culex, Anopheles* (due to their very large distribution, they were not considered)<br>→ Fish species: *Gambusia affinis, Oreochromis niloticus, Poecilia reticulata*<br>→ Marsupial Families: Didelphidae, Phalangeridae, Pseudocheiridae (since the MU model was trained on African data, where this taxonomic animal group does not exist, they were not considered)<br>→ Mammal species: *Thryonomys swinderianus*	33
	Forested areas	Globc40: closed to open (>15%) broadleaved evergreen and/ or semi-deciduous forest (>5m)	34
**PLANT DIVERSITY**	Diversity of native/introduced plants	Species richness and/or presence-absence by hexagon unit of 88×88km^2:<br>→ Family level: Aracaceae and Ipomoea.<br>→ Species level: *Commelina benghalensis*, *Echinochloa pyramidalis, Leersia hexandra,* and *Nymphaea lotus*	33
**SOCIO-DEMOGRAPHIC**	Agriculture	Equi_reg: Percentage of area equipped for irrigation	35
		Globc11 and Globc14: croplands	34
	Forest loss	Lossforest: non-intact forest in Africa (%)	36
	Human activity/presence	Denspobl: population density (person/Km^2)	37
		Distpop: distance to populated centres (m)	38
		Distrail: distance to railways (m)	39
		Distroad: distance to roads (m)	
	Human activity/presence	GRDIV1: Degree of deprivation of the population (0-100)	40
		SDH: Subnational human development index	
		VNL: Visible night lights at 2020	
		VNL_SLOPE: rate of change in night lights between 2012-2020	
Interactions		Two-way interaction terms were considered. Using three-way interactions would have complexified the statistical procedure	-
Logit equation		MU_model *logit*: *Mycobacterium ulcerans* model *logit* equation	Linear combinations of predictor variables that form part of the binomial-regression equations

Model fit assessment for Africa

We evaluated the discriminatory ability of the model by calculating the area under the Receiver Operating Characteristic (ROC) curve (AUC)[41]. In addition to the AUC, the model's classification accuracy was further assessed using the following six specific indices[42,43]: (1) sensitivity (i.e., proportion of the number of hexagons with positive cases correctly classified as favourable), (2) specificity (i.e., proportion of the number of hexagons with negative cases correctly classified as unfavourable), (3) Correct Classification Rate (i.e., proportion of hexagons, either with positive or negative cases, correctly classified), (4) True Skill Statistics (i.e., sensitivity + specificity - 1), which measures the overall classification performance of the model, (5) underprediction rate (i.e., proportion of unfavourable hexagons with positive cases), and (6) overprediction rate (i.e., proportion of favourable hexagons with negative cases).

The AUC values for the MU and BU models were 0.957 and 0.909, respectively, indicating 'outstanding' discrimination ability according to Hosmer and Lemeshow[44]. Sensitivity values indicated that over 85% of the hexagons with reported presence of MU and BU cases were correctly classified by the models, while underprediction rates were low (0.004 for MU and 0.0007 for BU, respectively). Thus, the occurrences of MU and reported BU cases for Africa were rarely underestimated. Specificity values ranged from 0.938 for MU to 0.881 for BU, respectively; thus from 6% to 11% of the hexagons initially labeled as 'absence' zones were predicted by the models to have favourable conditions. In both models, the True Skill Statistic (TSS) values were positive and >0.790. The Correct Classification Rate (CCR) values were 0.937 for the MU model and 0.882 for the BU model, respectively. Finally, overprediction values were 0.757 and 0.933. This means that the dispersion of presence data did not generate geographically dispersed favourability output estimates (see Figures 1 and 2), so these favourability models were accepted far from over-fitting.

Table S5 Model fit assessment based on discrimination and classification capacities respect to MU and BU records. TSS: true skill statistic, Sens.: sensitivity, Spec.: specificity, CCR: correct classification rate, Underp.: underprediction rate, Overp.: overprediction rate. AUC: area under the receiver operator characteristic curve.

	**TSS**	**Sens.**	**Spec.**	**CCR**	**Underp,**	**Overp.**	**AUC**
**MU model**	0.795	0.857	0.938	0.937	0.001	0.933	0.957
**BU model**	0.790	0.909	0.881	0.882	0.004	0.757	0.909

Table S6 Validation of model predictive capacity based on discrimination and classification performance respect to disease records. TSS, Sens., Spec., CCR, Underp., Overp., and AUC

	**Records of reference for validation**	**TSS**	**Sens.**	**Spec.**	**CCR**	**Underp,**	**Overp.**	**AUC**
**MU model**	BU cases	0.547	0.591	0.956	0.941	0.018	0.641	0.919

Supplementary References

1. Duker, A. A., Carranza, E. J. & Hale, M. Spatial dependency of Buruli ulcer prevalence on arsenic-enriched domains in Amansie West District, Ghana: implications for arsenic mediation in Mycobacterium ulcerans infection. *Int. J. Health Geogr.* **3**, 19 (2004).

2. de Thoisy, B. *et al.* Ecology, evolution, and epidemiology of zoonotic and vector-borne infectious diseases in French Guiana: Transdisciplinarity does matter to tackle new emerging threats. *Infection, Genetics and Evolution* **93**, 104916 (2021).

3. Keita, M. L. *et al.* Gold Panning–Related Chronic Cutaneous Ulcers in Guinea, West Africa. *Am. J. Trop. Med. Hyg.* **108**, 221–226 (2022).

4. Guégan, J.-F. *et al.* Canonical fact versus hypothesis testing to decipher transmission of non-tuberculous and tuberculous mycobacteria: a comparative review. *Clinical Microbiology Reviews, in review* (2025).

5. Reith, F. *et al.* Mechanisms of gold biomineralization in the bacterium *Cupriavidus metallidurans*. *Proceedings of the National Academy of Sciences* **106**, 17757–17762 (2009).

6. Honda, J. R., Virdi, R. & Chan, E. D. Global Environmental Nontuberculous Mycobacteria and Their Contemporaneous Man-Made and Natural Niches. *Front. Microbiol.* **9**, (2018).

7. Stone, W., Kroukamp, O., Korber, D. R., McKelvie, J. & Wolfaardt, G. M. Microbes at Surface-Air Interfaces: The Metabolic Harnessing of Relative Humidity, Surface Hygroscopicity, and Oligotrophy for Resilience. *Front. Microbiol.* **7**, (2016).

8. Zhou, X. *et al.* Global analysis of soil bacterial genera and diversity in response to pH. *Soil Biol. Biochem.* **198**, 109552 (2024).

9. Morris, A. *et al.* First Detection of Mycobacterium ulcerans DNA in Environmental Samples from South America. *PLoS Negl. Trop. Dis.* **8**, e2660 (2014).

10. Garchitorena, A. *et al.* Mycobacterium ulcerans dynamics in aquatic ecosystems are driven by a complex interplay of abiotic and biotic factors. *Elife* **4**, (2015).

11. Sanhueza, D., Chevillon, C., Bouzinbi, N., Godreuil, S. & Guégan, J.-F. Chitin Increases Mycobacterium ulcerans Growth in Acidic Environments. *Microbes Environ.* **33**, 234–237 (2018).

12. Mod, H. K. *et al.* Predicting spatial patterns of soil bacteria under current and future environmental conditions. *ISME J.* **15**, 2547–2560 (2021).

13. Kumar, R., Kumar, K. & Thakur, N. Biosynthesis of CuO/Cu2O-ZnO nanocomposites via Commelina benghalensis leaf extract and their antibacterial, photocatalytic and antioxidant assessment. *Inorg. Chem. Commun.* **157**, 111400 (2023).

14. Doolabh, K. *et al.* Phytochemical composition and potential antibacterial activity of the tropical spiderwort (Commelina benghalensis L.). *Appl. Ecol. Environ. Res.* **23**, 1257–1279 (2025).

15. Akpanudo, N. W. & Olabemiwo, O. M. Green synthesis and characterization of copper nanoparticles (CuNPs) and composites (CuC) using the *Echinochloa pyramidalis* extract and

their application in the remediation of PAHsin water. *Water Pract. Technol.* **19**, 324–342 (2024).

16. Hintsho-Mbita, N. C., Maeko, M., Mathipa, M. M. & Tetana, Z. N. Photodegradation of methylene blue dye and deactivation of E.coli and S.aureus using C.benghalensis zinc ferrite nanoparticles. *Desalination Water Treat.* **321**, 101003 (2025).
17. Asongwe, G. A. *et al.* Influence of urbanisation on phytodiversity and some soil properties in riverine wetlands of Bamenda municipality, Cameroon. *Sci. Rep.* **12**, 19766 (2022).
18. Simpson, H. *et al.* Mapping the global distribution of Buruli ulcer: a systematic review with evidence consensus. *Lancet Glob. Health* **7**, e912–e922 (2019).
19. Hammoudi, N. *et al.* Tracing Mycobacterium ulcerans along an alimentary chain in Côte d'Ivoire: A one health perspective. *PLoS Negl. Trop. Dis.* **14**, e0008228 (2020).
20. Pileggi, S. M. *et al.* Landscape and environmental influences on Mycobacterium ulcerans distribution among aquatic sites in Ghana. *PLoS One* **12**, e0176375 (2017).
21. Vandelannoote, K. *et al.* Multiple introductions and recent spread of the emerging human pathogen *Mycobacterium ulcerans* across Africa. *Genome Biol. Evol.* evx003 (2017) doi:10.1093/gbe/evx003.
22. Brou, T., Broutin, H., Elguero, E., Asse, H. & Guegan, J.-F. Landscape Diversity Related to Buruli Ulcer Disease in Côte d'Ivoire. *PLoS Negl. Trop. Dis.* **2**, e271 (2008).
23. Marion, E. *et al.* Buruli Ulcer Disease in Republic of the Congo. *Emerg. Infect. Dis.* **20**, 1070–1072 (2014).
24. Bayonne Manou, L. S. *et al.* Mycobacterium ulcerans disease (Buruli ulcer) in Gabon: 2005-2011. *Med. Sante Trop.* **23**, 450–457 (2013).
25. Schweitzer, P. N. Record quality tables for the Mineral Resources Data System: U.S. https://mrdata.usgs.gov/mrds/ (2019) doi:https://doi.org/10.5066/P9DYLWMP.
26. Shangguan, W., Dai, Y., Duan, Q., Liu, B. & Yuan, H. A Global Soil Data Set for Earth System Modeling. *J. Adv. Model. Earth Syst.* http://globalchange.bnu.edu.cn/research/soilw#download (2014).
27. US Geological Survey. GTOPO30. https://lta.cr.usgs.gov/GTOPO30 (1996).
28. Center for Global Environmental Research. Global Drainage Basin Database GDBD. Released version 1.0: May 29, 2007. http://www.cger.nies.go.jp/db/gdbd/gdbd_index_e.html (2007).
29. Spawn, S. A., Sullivan, C. C., Lark, T. J. & Gibbs, H. K. Harmonized global maps of above and belowground biomass carbon density in the year 2010. *Sci. Data* **7**, 112 (2020).
30. FAO soils portal. Harmonized World Soil Database v2.0. https://www.fao.org/soils-portal/data-hub/soil-maps-and-databases/harmonized-world-soil-database-v20/en/ (2023).
31. Karger, D. N. , *et al.* Climatologies at high resolution for the earth's land surface areas. *EnviDat* https://doi.org/10.16904/envidat.228. (2021) doi:10.16904/envidat.228.
32. Beckmann, M. *et al.* glUV: a global UV-B radiation data set for macroecological studies. *Methods Ecol. Evol.* **5**, 372–383 (2014).
33. Gbif.org. GBIF Occurence download. Accessed 06 February 2025. (2025).

34. Food and Agriculture Organization of the United Nations (FAO). GlobCover land cover maps. Version 2.3 database. https://www.fao.org/land-water/land/land-governance/land-resources-planning-toolbox/category/details/en/c/1036356/ (2009).

35. Food and Agriculture Organization of the United Nations (FAO). Global Map of Irrigation Areas (version 4.0.1) around the year 2000. https://www.fao.org/land-water/water/en.

36. Hansen, M. C. *et al.* High-Resolution Global Maps of 21st-Century Forest Cover Change. *Science (1979).* **342**, 850–853 (2013).

37. Salvatore, M., Pozzi, F., Ataman, E., Huddleston, B. & Bloise, M. *Mapping Global Urban and Rural Population Distributions*. (FAO, Rome, 2005).

38. Food and Agriculture Organization of the United Nations (FAO). Administrative Centres & Populated Places shapefile at the Relational World Database II (RWDB2). https://data.apps.fao.org/map/catalog/srv/eng/catalog.search#/metadata/93e9ab90-b06d-11db-8922-000d939bc5d8 (2005).

39. Harvard University. Vector map level 0. http://worldmap.harvard.edu. (2002).

40. Center for International Earth Science Information Network (CIESIN)). Global Gridded Relative Deprivation Index (GRDI), Version 1. *Columbia Climate School* https://storymaps.arcgis.com/stories/2012e4b3c51c476e858b04fbec0e8cd2 (2023).

41. Lobo, J. M., Jiménez-valverde, A. & Real, R. AUC: A misleading measure of the performance of predictive distribution models. *Global Ecology and Biogeography* **17**, 145–151 (2008).

42. Barbosa, A. M. & Real, R. Applying fuzzy logic to comparative distribution modelling: a case study with two sympatric amphibians. *The Scientific World Journal* **2012**, 1–10 (2012).

43. Fielding, A. H. & Bell, J. F. A review of methods for the assessment of prediction errors in conservation presence/absence models. *Environ. Conserv.* **24**, 38–49 (1997).

44. Hosmer, D. W. & Lemeshow, S. *Applied Logistic Regression*. *Applied Logistic Regression* (John Wiley & Sons, Inc., Hoboken, New York, 2000). doi:10.1002/0471722146.
